# Structure and function of a near fully-activated intermediate GPCR-Gαβγ complex

**DOI:** 10.1038/s41467-025-56434-4

**Published:** 2025-01-28

**Authors:** Maxine Bi, Xudong Wang, Jinan Wang, Jun Xu, Wenkai Sun, Victor Ayo Adediwura, Yinglong Miao, Yifan Cheng, Libin Ye

**Affiliations:** 1 Department of Biochemistry and Biophysics, University of California, 600 16th Street, San Francisco, CA 94143 USA; 2 Department of Molecular Biosciences, University of South Florida, 4202 E Fowler Ave, Tampa, FL 33620 USA; 3 Pharmacology & Computational Medicine Program, University of North Carolina at Chapel Hill, 116 Manning Drive, Chapel Hill, NC 27599 USA; 4 Department of Molecular and Cellular Physiology, Stanford University School of Medicine, Stanford, CA USA; 5 Howard Hughes Medical Institute, University of California, 600 16th Street, San Francisco, CA 94143 USA; 6 H. Lee Moffitt Cancer Center & Research Institute, 12902 USF Magnolia Drive, Tampa, FL 33612 USA

**Keywords:** Cryoelectron microscopy, G protein-coupled receptors, Solution-state NMR

## Abstract

Unraveling the signaling roles of intermediate complexes is pivotal for G protein-coupled receptor (GPCR) drug development. Despite hundreds of GPCR-Gαβγ structures, these snapshots primarily capture the fully activated complex. Consequently, the functions of intermediate GPCR-G protein complexes remain elusive. Guided by a conformational landscape visualized via ^19^F quantitative NMR and molecular dynamics (MD) simulations, we determined the structure of an intermediate GPCR-mini-Gα_s_βγ complex at 2.6 Å using cryo-EM, by blocking its transition to the fully activated complex. Furthermore, we present direct evidence that the complex at this intermediate state initiates a rate-limited nucleotide exchange before transitioning to the fully activated complex. In this state, BODIPY-GDP/GTP based nucleotide exchange assays further indicated the α-helical domain of the Gα is partially open, allowing it to grasp a nucleotide at a non-canonical binding site, distinct from the canonical nucleotide-binding site. These advances bridge a significant gap in our understanding of the complexity of GPCR signaling.

## Abstract (web summary)

Here, the authors employed 19 F NMR and Gaussian accelerated MD simulations to determine the conformational landscape of the adenosine-A2A receptor and mini-Gαsβγ complex, enabling determination of an intermediate structure of the complex with cryoEM.

## Introduction

G protein-coupled receptors (GPCRs) are the largest family of human membrane proteins, encompassing over 800 distinct members. Due to their vital roles in various (patho) physiological processes, significant efforts have been made to unravel the processes of their activation, aiming to modulate therapeutic signaling with precision. Recent advancements in X-ray crystallography and single-particle cryo-electron microscopy (cryo-EM) have facilitated the structural determination of hundreds of GPCR-Gαβγ complexes since 2017^1–3^. These structures primarily represent the receptors in their fully activated complexes. However, the GPCR signaling process is more intricate than what these structures alone can depict. Multiple studies have shown that GPCR activation entails transitions through several intermediate complexes, including pre-coupled and partially activated GPCR-G protein complexes^4–12^. Yet, isolating such transient complexes to study their signaling roles presents a significant challenge, as it is nearly impossible to biochemically isolate GPCRs in these intermediate states. Characterizing structural and functional roles of individual intermediate GPCR-G protein complexes will significantly advance our knowledge at the molecular mechanistic level regarding signaling efficacy, bias, and allostery, which are pivotal for improving drug designs^5,13,14^.

**Fig. 1:**
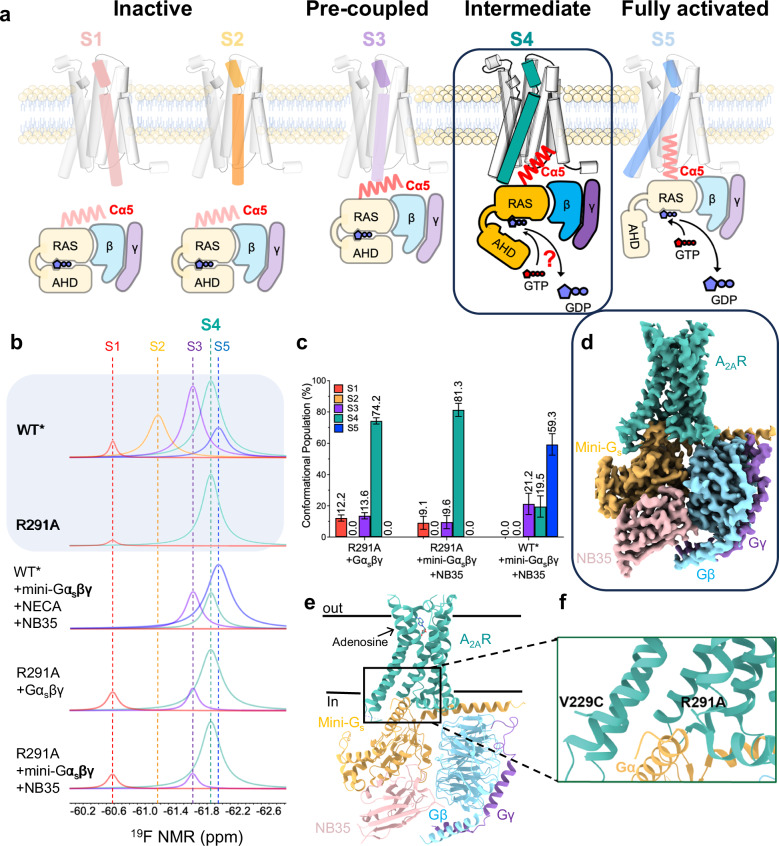
Identification of the intermediate complex investigated in this study. **a** Proposed activation model of GPCR, including inactive states S1 and S2, pre-coupled complex (S3-Gα_s_βγ), intermediate complex (S4-Gα_s_βγ), and the fully activated complex (S5-Gα_s_βγ), in which S4-Gα_s_βγ was highlighted in teal for this study. **b** In reference to Supplementary Fig. 1, the deconvoluted conformational profiles probed by^19^F-qNMR for the R291A construct were presented as a function of Gα_s_βγ and mini-Gα_s_βγ. ^19^F-qNMR spectra of WT* and R291A were used as the benchmarks for S1 through S5, adapted from the reference (Wang et al, 2023, Nature Commun). The spectrum of WT*+mini-Gα_s_βγ + NECA + NB35 represents the feature for PDB: 6GDG. **c** The population distributions of conformational states for R291A+mini-Gα_s_βγ+NB35, R291A + Gα_s_βγ, and WT*+mini-Gα_s_βγ+NB35 (S5-Gα_s_βγ). The source data for these population distributions are included in the Source Data file. Data with error bars are presented as state population±SD. The SD values were determined based on spectral S/Ns and fitting errors of the deconvolutions. **d** Cryo-EM density map of the intermediate S4-mini-Gα_s_βγ complex. **e** Ribbon representation of S4-mini-Gα_s_βγ. **f** Highlighted interfacial section of S4-mini-Gα_s_βγ.

Here, using the adenosine-A_2A_ receptor (A_2A_R) as a model system and applying ^19^F quantitative NMR (^19^F-qNMR), we visualize the conformational landscape of this GPCR in response to ligand actions and transducers such as G proteins. We identified two transient active-like intermediates (S3 and S4) that are positioned between the inactive (defined as S1 and S2) and the fully activated states (S5)^15^ (Fig. 1a). Among these conformations, S1 and S2 have been defined by crystal structures^16,17^, and the S5 state has been characterized by cryo-EM (PDB ID: 6GDG)^18^ (Fig. 1b, c, and Supplementary Fig. 1). The structures of S3 and S4, however, remain uncharacterized. With the R291A point mutation, which we previously identified as trapping A_2A_R in the intermediate S4 state (Fig. 1)^15^, we investigate the structure and function of this intermediate state. We demonstrate that intermediate S4 can directly interact with and regulate G protein for a rate-limited nucleotide exchange prior to transitioning to the S5 state. We also determine the structures of A_2A_R at S4 in complex with mini-Gα_s_βγ, revealing how the G protein engages with the intermediate S4. Further, biochemical assessments and Gaussian accelerated MD (GaMD) simulations unravel a mechanism in which the intermediate GPCR-G protein complex not only exhibits a limited GTP turnover capacity but also elicits a slower GTP-GDP exchange prior to transitioning to the fully activated complex. Beyond A_2A_R, our approach can be applied to characterize the functions and structures of transient intermediate states and their complexes in other GPCRs.

## Results

### S4 state binds to Gα_s_βγ and initiates nucleotide exchange

We first examined whether the WT*-R291A mutant, which is trapped in the S4 state, can bind to the Gα_s_βγ. Here, the S4 trapping mutant was introduced to a previously defined construct (A_2A_R-316-V229C, denoted as WT*)^15^, and we referred to WT*-R291A simply as R291A. We incubated ^19^F-labeled R291A with Gα_s_βγ, subjected it to ^19^F-NMR, and measured the linewidth of the NMR resonance. Indeed, we observed linewidth broadening for the S4 resonance compared to the receptor alone, suggesting direct binding of Gα_s_βγ to A_2A_R in the S4 state (Supplementary Fig. 2a). This was further confirmed by a complex band of R291A-Gα_s_βγ in native-PAGE (Supplementary Fig. 2b) without the addition of an exogenous ligand.

**Fig. 2:**
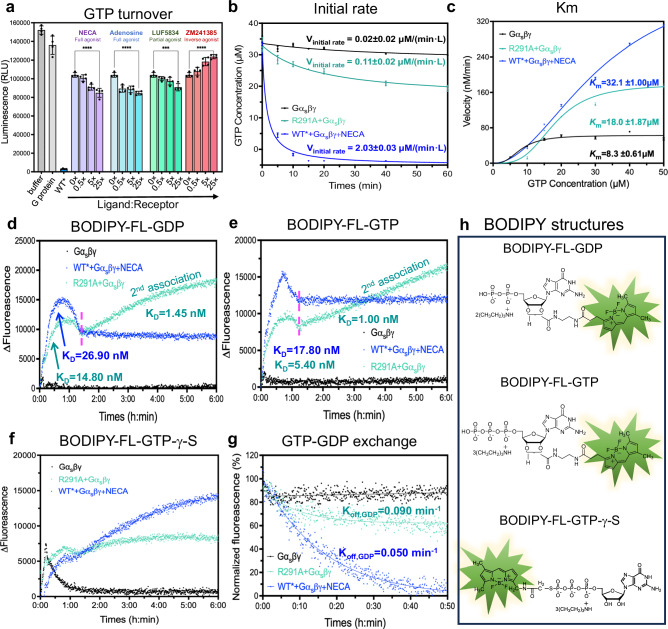
S4 mediated GTP hydrolysis and nucleotide exchange. **a** GTP hydrolysis of the S4 mediated Gα_s_βγ as a function of inverse, partial, and full agonists, in reference to Gα_s_βγ alone (negative control) and the S5 mediated Gα_s_βγ (positive control). Data with error bars are presented as mean ± SEM of four independent experiments. Statistical analyses were performed using the ordinary one-way ANOVA compared to the R291A apo, ***p < 0.001, and ****p < 0.0001. **b** Time course of GTP hydrolysis of the S4 mediated Gα_s_βγ, in reference to Gα_s_βγ alone (negative control) and the S5 mediated Gα_s_βγ (positive control); the initial rate of each catalysis was calculated. The initial rate of each catalysis was calculated. Data are presented as mean values ± SD from three independent experiments (*n* = 3). Source data are provided in the Source Data file. **c** Km values for S4 mediated Gα_s_βγ, in reference to Gα_s_βγ alone (negative control) and the S5 mediated Gα_s_βγ (positive control). Data are presented as mean values ± SD from three independent experiments (*n* = 3). Source data are provided in the Source Data file. **d** BODIPY-FL-GDP binding assay. **e** BODIPY-FL-GTP binding assay. **f** BODIPY-FL-GTP-γ-S binding assay (**g**) GTP-GDP exchange rate comparison. **h** The structures of BODIPY-FL-GDP, BODIPY**-**FL-GTP, and BODIPY-FL-GTP-γ-S.

Next, we assessed how the S4 state regulates G protein function by performing nucleotide hydrolysis, binding, and exchange assays. As shown in Fig. 2a, the R291A mutant-mediated GTP hydrolysis from the G protein occurred at a much slower rate compared to the full agonist-bound WT*-Gα_s_βγ (representing the S5-Gα_s_βγ). However, the additions of full, partial, or inverse agonists did not alter the GTP hydrolysis level substantially in the R291A mutant. This ligand-independent hydrolysis behavior of the R291A mutant is in stark contrast to the wild-type construct, where ligand binding dramatically changes the GTP hydrolysis level^19,20^, suggesting that the R291A mutation indeed traps the complex in this intermediate state for characterization. To further investigate the reduced hydrolysis level, we measured the initial rates and Michaelis constants of GTP hydrolysis. The initial rate of GTP hydrolysis mediated by R291A was only one-twentieth of that mediated by WT* (Fig. 2b), while the Michaelis constant exhibited a similar pattern (Fig. 2c).

We proposed a simple model to explain the limited GTP hydrolyzing capacity of the S4-mediated G protein. In the S4-G protein complex, nucleotide GTP binds to a noncanonical location (site 2) with a limited space formed by Ras-like domain and α-helical domain (AHD) resulting from a partial opening of AHD. This pose of G protein also exhibited a limited GTPase activity, along with nucleotide exchange. Upon transitioning from S4 to S5, the G protein changes its conformation to allow the GTP to access site 1, the canonical nucleotide-binding site. We tested this model by examining the binding of BODIPY-FL-GDP (Fig. 2d and h). The BODIPY-FL-GDP bound to the WT*-mediated G protein exhibited a higher K_D_ than that of the R291A-mediated G protein, although both reached equilibrium at 1.5 hrs. For the R291A-mediated G protein, a secondary association event was observed after 1.5 hrs with a K_D_ value 10 times lower, which was not the case observed in the WT*. We attribute this difference to the restricted accessibility of site 1, caused by spatial constraints resulting from the partially open AHD in the S4-mediated G-protein, while this site is freely accessible in the WT*. A similar pattern was noted in BODIPY-FL-GTP binding assays, where BODIPY-FL-GTP showed more efficient binding to the S5-mediated G protein with a higher K_D_ value (Fig. 2e and h). The spatially limited association process was also evident in BODIPY-FL-GTP-γ-S (Fig. 2f) in both S5 and S4 mediated G protein because the BODIPY head was linked to the γ-phosphate, which is meant to be inserted into site 1. The bulky BODIPY head insertion restricted exchange and hydrolysis due to the space limitation, supporting our hypothesis that the noncanonical binding site 2 was formed by a not fully opened AHD. Titration of GTP into the BODIPY-FL-GDP equilibrium system led us to conclude that the release number of BODIPY-FL-GDP from S4-mediated G protein is much less than the S5-mediated G protein (Fig. 2g and Supplementary Fig. 3) but reach the equilibrium much faster with the K_off_ of 0.09/min vs 0.05/min. A previous study of β_2_AR indicated that GDP release occurs much faster than the formation of the fully activated complex^21^, while MD simulations suggested that GDP is released when the Gα_s_ is not fully opened^22^. Collectively, our data suggest that the intermediate S4 state-mediated G-protein initiates nucleotide exchange before transitioning to the fully activated S5 state, albeit at a reduced rate.

### Structures of the intermediate

#### S4-mini-Gα_s_βγ complex

Building upon the two-step nucleotide binding model described above, we hypothesized that the S4 state adopts a conformation distinct from S5, interacting with the G protein differently from the fully activated S5-bound state. To test this hypothesis, we determined the structures of the intermediate complex trapped in the S4 state. A stable complex was assembled by incubating the R291A mutant with mini-Gα_s_ (mini-Gα_s_399) and the Gα_s_-stabilizing nanobody, NB35, along with Gβ_1_γ_2_ (Supplementary Fig. 4). As shown by ^19^F-qNMR, the R291A mutation enriches the population of the S4 state with minimal variations (Fig. 1b, c). A ligand density seen in the ligand binding pocket is interpreted as endogenous adenosine since no exogenous ligand was added. Mass spectrum further confirms the existence of endogenous adenosine in the complex (Supplementary Fig. 5). Thorough 3D variability analysis (3DVA) (Supplementary Movie 1) and rigorous 3D classifications captured a predominant cryo-EM structure of the S4-mini-Gα_s_βγ complex at 2.6 Å resolution (Supplementary Figs. 6–8). In addition to this predominant S4 structure, two dynamic snapshots, referred to as S4_d1_ and S4_d2_, were identified. This is consistent with the ^19^F-qNMR profiles, which suggest a degree of conformational dynamics within the S4 state, as indicated by a broad resonance. Together, these structures reveal dynamic behaviors of A_2A_R at the S4 state, providing insight into key interactions between the receptor and mini-Gα_s_βγ (Fig. 1d–f).

**Fig. 3:**
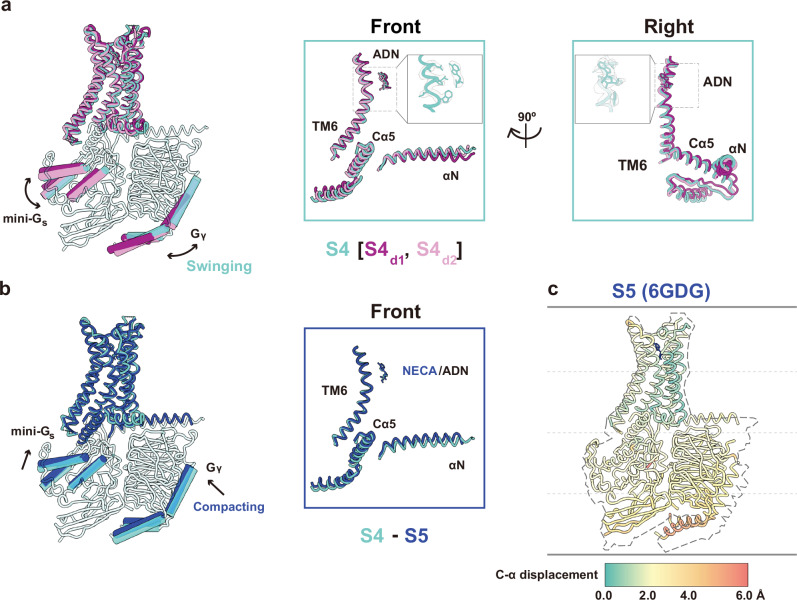
Overlay of S4 [S4_d1_, S4_d2_] and S5 states showing intermediate transitions. **a** Superimposed licorice representations of S4 [S4_d1_, S4_d2_]-mini-Gα_s_βγ aligned by TM1, with key motions highlighted using cylinders. The front view illustrates a slight shift in adenosine binding and key transitions near TM6, Cα5, and αN helices, also shown in the right side view. **b** Superimposed licorice representations of S5, S4 [S4_d1_, S4_d2_]-mini-Gα_s_βγ, with key motions highlighted using cylinders. The front view focuses on transitions near TM6, Cα5, and αN helices. **c** C-α displacement representation of the S5 state, with a dashed outline indicating the corresponding S4 state for reference. The C-α displacement color bar, mapped to each C-α atom, ranges from 0.0 Å (teal) to 6.0 Å (red), with intermediate values at 2.0 Å (light yellow) and 4.0 Å (peach).

Visual comparison between these snapshots suggests that S4_d1_ and S4_d2_ represent dynamic variations of the predominant S4 conformation, which we simply refer to as S4 conformation, with minimal changes in the receptor and its engagement with G protein, but noticeable swinging motions of the G protein (Fig. 3a and Supplementary Movie 2). To further assess this, we calculated the displacement of each alpha carbon (C_α_) atom between the S4 state and these dynamic snapshots. Structural changes in S4_d1_ and S4_d2_ reached up to 2 Å, primarily near the TM6 region. While the Cα5 helix of mini-Gα_s_ which engages the receptor, remained stable, the αN helix and the outermost portion of the G protein exhibited more pronounced shifts. The most significant movement was observed in the Gγ subunit in the S4_d1_ state relative to the S4 state (Supplementary Fig. 9). In the S4 conformation, the G protein aligns more closely with the receptor’s center. This suggests that in the S4 state, the G protein may have weaker engagement with the receptor compared to S5, allowing for some swinging motion around the predominate orientation.

Despite these deviations, most particles aligned with the S4 conformation, indicating that the majority are stabilized in this state. Given the predominance of the S4 state, we designate this conformation as the S4 and focus our structural comparison on the transition from S4 to S5. As shown in Fig. 3b, the final step in this transition involves compacting the G protein into a more engaged conformation, completing the insertion process. C_α_ displacement calculations between the S4 and S5 states (Fig. 3c) revealed deviations of up to 6 Å at the Gγ subunit, reflecting significant conformational changes during the final steps of insertion. These results suggest that the S4 state, along with its dynamic snapshots, aligns with a broader trajectory toward the fully activated S5 state.

**Fig. 4:**
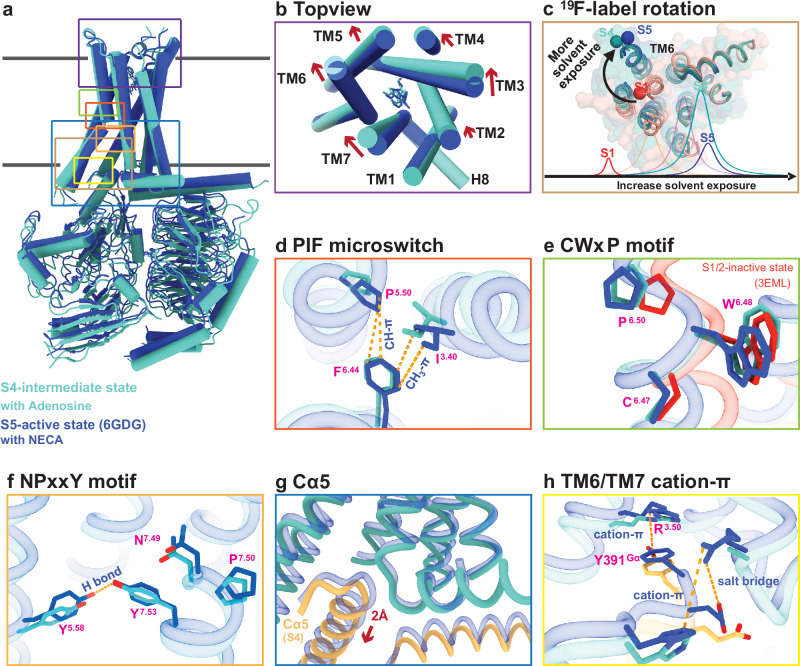
Structural comparison of the receptor in the intermediate S4 and fully activated S5 states. **a** Superimposed cylinder representations of S4-mini-Gα_s_βγ and S5-mini-Gα_s_βγ. **b** Top view of ligand binding domain of the S4 state compared to the S5 state. **c** ^19^F-tag rotation, and corresponding NMR spectrum. **d** PIF microswitch. **e** CWxP motif. **f** NPxxY motif. **g** Cα5 motion of Gα_s_. **h** TM6 and TM7 cation-π interaction in two complexes.

Comparisons of the S4-mini-Gα_s_βγ complex with the S5-mini-Gα_s_βγ complex (PDB ID: 6GDG) reveal critical differences between these states (Fig. 4a). Specifically, the extracellular orthosteric binding pocket of the intermediate S4-mini-Gα_s_βγ complex is less compact than that of the S5-mini-Gα_s_βγ (Fig. 4b), yet more constricted compared to the inactive state^16,17^. Transitioning from the inactive to the intermediate S4 state involves significant inward movements of all domains relative to TM1 in the extracellular regions. In contrast, the transition from the intermediate S4 state to the fully activated S5 state, though involving smaller and more refined movements, plays an important role in the final stages of activation. These subtle but significant adjustments include a 2 Å clockwise rotation of TM5 and TM6 and a partial to full insertion of the G protein. Despite their smaller scale, these movements are essential for stabilizing the active conformation and ensuring full activation of the G protein. This is consistent with the ^19^F-qNMR study, which recorded a substantial chemical shift (~400 Hz) when the receptor transitioned from the inactive states S1-2 to intermediate S4, while the shift from S4 to S5 resulted in a smaller chemical shift (~60 Hz). Given the subtle change between S4 and S5, we define S4 as a nearly fully-activated intermediate state, distinct from other intermediate states that will be studied in the future. Closer inspection of the TM6 domain, using the ^19^F-tag labeling site as a reference, revealed a clockwise rotation consistent with the ^19^F-qNMR resonance, where the NMR signal for the S4 state is at a lower field than that of the S5 state (Fig.4c).

One of the key microswitches in the receptor is the P^5.50^I^3.40^F^6.44^ motif, which is a crucial hydrophobic switch between TM5, TM3, and TM6 and is implicated in receptor activation^1,23^. In the fully activated receptor, P^5.50^ and F^6.44^ are in cis positions, forming a strong CH-π interaction^24^, and the CH_3_ group of I^3.40^ also forms a robust CH_3_-π interaction with F^6.44^, stabilizing the fully activated conformation. In contrast, these interactions are weakened in the S4-mini-Gα_s_βγ complex because P^5.50^ and I^3.40^ are oriented away from F^6.44^ (Fig. 4d). Another key microswitch is the CW^6.48^xP motif, where P^6.50^ acts as a hinge in TM6 and facilitates the opening of the cytoplasmic cavity during activation^25^. Our cryo-EM structure clearly shows P^6.50^ in an “intermediate” position, bridging the inactive and active states (Fig. 4e). The third microswitch, the NPxxY^7.53^ on TM7, along with a conserved tyrosine Y^5.58^ in TM5, stabilizes the receptor’s active state^26–28^. As illustrated in Fig. 4f, a strong H-bond is observed between Y^5.58^ and Y^7.53^ in the S5-mini-Gαβγ complex. This interaction draws Y^5.58^ on TM5 closer to TM7, maintaining the compactness of the TM bundle in the S5-G protein complex, as shown in the animation from the S4-mini-Gα_s_βγ transitioning to the S5-mini-Gαβγ (Supplementary Movie 3). Transitioning from the S4- to the S5-mini-Gα_s_βγ strengthens the H-bond interaction as Y^5.58^ moves closer to the TM7 domain, facilitating the insertion of the G protein into the cavity.

Next, we examined the interfacial interaction between the Cα5 helix of mini-Gα_s_ (Fig. 4g) and the transmembrane bundle comprising TM3, TM6, and TM7 domains, which are critical components in regulating G protein nucleotide exchange. As highlighted in Fig. 4g, the S4-mini-Gα_s_βγ adopts a less compact conformation than the S5-mini-Gα_s_βγ. The Cα5 helix is rotated outward and retracted from the G protein-binding pocket, accompanied by the disengagement of the αN helix of the Gα_s_ protein from the H8 helix of the receptor. This retraction measures ~2 Å, and the rotation is at a 2° spinal clockwise angle from the S5-mini-Gα_s_βγ. This motion is further detailed in Fig. 4h, which shows the primary interactions at the TM3, TM6, and TM7 junction with the Cα5 segment from the Gα_s_, including the formation of an intermolecular salt bridge R^7.56^-E392^Gα^ and the cation-π interaction between R^7.56^ and H^6.32^, along with a strengthened cation-π interaction between R^3.50^ and Y391^Gα^. However, the R^7.56^ to A^7.56^ mutation disrupts these interactions. During activation, the clockwise rotation of TM6 and counterclockwise rotation of Cα5 culminate in the fully activated S5 complex. The interactions observed in the S4-mini-Gα_s_βγ represent an intermediate stage in this process (Supplementary Movie 4). Our structural analysis reveals distinct engagements between the receptor and G protein in the S4 and S5 states, which may help account for the slower rate of GTP hydrolysis in the S4 state. This suggests that the S4 conformation can modulate G protein signaling without fully transitioning to the S5 state.

### Molecular basis of A_2A_R function in the S4 state

**Fig. 5:**
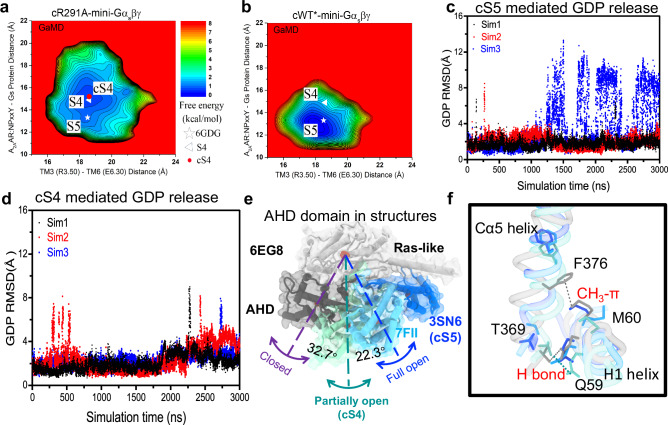
Structural features of A_2A_R and G protein in GaMD simulation. **a** 2D free energy profiles of the cR291A-mini-Gα_s_βγ complex system. **b** 2D free energy profiles of the cWT*-mini-Gα_s_βγ complex systems. The white triangle and asterisk indicate cryo-EM structures of the S5 and S4 states, respectively. **c** Root-mean square deviation (RMSD) of GDP relative to the initial position in the GaMD simulation for cS5 mediated G protein. **d** RMSD of GDP relative to the initial position in the GaMD simulation for cS4 mediated G protein. **e** The positions of AHD when G protein is inactive, partial activated, and fully activated. **f** Superimposed positions of Cα5 and H1 helices when G protein is inactive, partial activated, and fully activated.

To validate the conformational transition between the S4 and S5 states, we applied all-atom Gaussian accelerated MD (GaMD) simulations, starting from the atomic structure of the S5-mini-Gα_s_βγ complex (6GDG) with an R291A mutation modeled in silico. Indeed, the R291A mutation facilitated the transition of the 6GDG structure (S5 state) to an energy minimum conformation that closely matches our cryo-EM structure (Fig. 5a). We refer to this as the cS4 state, where “c” stands for computational model, and here the Cα5 helix in Gα_s_ retracted from the receptor by ~2 Å. The center-of-mass distance between the receptor NPxxY motif and the last five residues of Cα5 helix increased from ~13.2 Å in the “S5” state to ~15.3 Å in the “cS4” state (Fig. 5a and Supplementary Fig. 10). As a control, GaMD simulations showed that the cWT-mini-Gα_s_βγ complex remained stable, mainly sampling one low-energy state, corresponding to the S5 (Fig. 5b). These simulations independently validated the conformation of the intermediate S4 state determined from single particle cryo-EM.

Furthermore, we performed additional GaMD simulations to examine GDP release. In the cWT-Gα_s_βγ system, GDP was observed moving away from the initial binding site by up to ~12 Å repeatedly (Fig. 5c). In contrast, GDP underwent significantly smaller movements up to only ~8 Å in the cR291A-Gα_s_βγ system (Fig. 5d). Free energy calculations also indicated a “GDP Released” state in the cWT-Gα_s_βγ system and only a “Partially Released” state in the cR291A-Gα_s_βγ system (Supplementary Fig. 11d, e), where the AHD of Gα_s_ transitioned from the “Open” to “Partially Open” conformation, with the orientation angle between the AHD and Ras-like domain decreasing to ~30° (Supplementary Fig. 11a–c)^1,29^. Together, these observations suggest that the S4 state mediated G protein has a reduced capacity for GDP release compared to the S5 state, aligning with the nucleotide exchange data shown above.

### A limited nucleotide exchange model for the intermediate GPCR-Gα_s_βγ complex

**Fig. 6:**
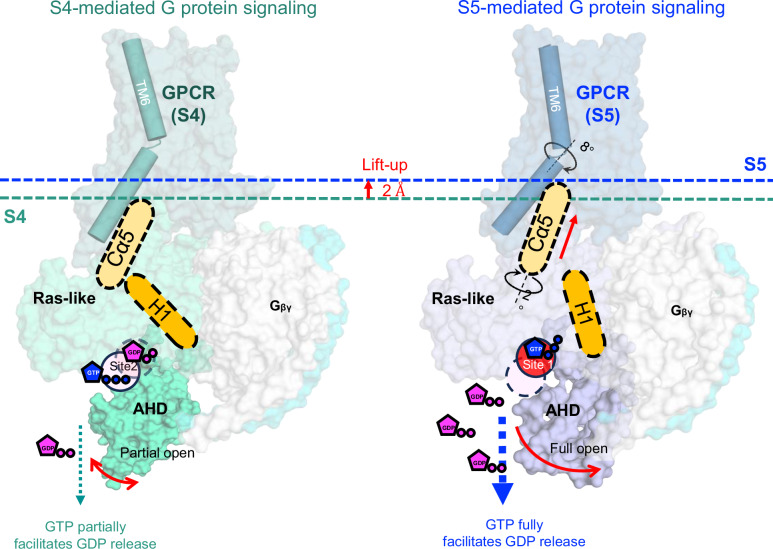
A limited nucleotide exchange model for intermediate GPCR-Gα_s_βγ complex. In the S5-mediated Gα the interaction between Cα5 and H1 helices was interrupted while this interaction is partially maintained through a weakened H-bond between Cα5 and H1 helices in Ras-like domain.

A mechanistic model is proposed to explain the rate-limited nucleotide exchange in this near fully-activated intermediate GPCR-G protein complex (Fig. 6). The transition from the near fully-activated intermediate to the fully activated complex involves a conformational change at the interface, where the TM6 helix of the receptor rotates clockwise by 8°, and the Cα5 helix from the Ras-like domain rotates anticlockwise by 2°. These movements result in a more compact interaction between the receptor and G protein, along with a 2 Å uplift of the Cα5 helix. This uplift disengages H1 from the Cα5 helix, facilitating the separation of the AHD from the Ras-like domain and thereby exposing the bound GDP at site 1. Simultaneously, this process supports the relocation of GTP from site 2 to site 1, replacing GDP. However, in the S4-mediated G protein, the substitution of R^7.56^ with alanine (A^7.56^) leads to the loss of both the intramolecular cation-π (R^7.56^-H^6.32^) interaction and the intermolecular salt bridge (R^7.56^-E392^Gα^). Instead, a partially open AHD is observed (Fig. 5e), which can be attributed to a weaker electrostatic H-bond (Q59-T369, 4 Å)^30^ between the Cα5 and H1 helices. In contrast, in the closed state of the AHD, an additional CH_3_-π interaction between F376 and M60 plays a significant role (Supplementary Fig. 12a–c). Notably, among all complex structures resolved so far, the crystallographic structure of the β2AR-Gαβγ complex is the only fully activated structure, in which the AHD opens by 88° whereas in other complexes, the opening is between 55 and 65°. This may be an effect of crystallographic packing during the crystallization process^31^. Collectively, these findings provide a molecular basis for the limited nucleotide exchange observed in the intermediate GPCR-G protein complex.

## Discussion

Previously, partially activated states of the sole A_2A_R receptor stabilized by partial agonists of LUF5833 and LUF5834 in the absence of G proteins, were resolved with the help of BRIL-fusion protein thermos-stabilization^32,33^. When comparing these two structures with the receptor portion in our complex, it is evident that the TM5 and TM6 domains are more open in our structure, supporting the idea that G protein binding at the intracellular domain further drives receptor activation (Supplementary Fig. 13)^34^. Consequently, the S4-mini-Gα_s_βγ represents a near fully-activated intermediate GPCR-G protein complex, transitioning toward the fully activated complex. This transition is halted due to the loss of a key intermolecular interaction between the residue R291 from the R291A mutant and E328 from the G protein_Gs_, a salt bridge essential for lifting the Ras-like domain of Gα_s_ to disengage the AHD.

Previous MD simulations have shown that GTPγS and GDP exhibit distinct contact patterns with G protein^35^. A recent study also demonstrated a 2 Å transition of the GTP nucleotide in a β_2_AR-regulated G protein, shifting from a “loose” interaction site to the final nucleotide-binding pocket^31,36^. Our study provides evidence of nucleotide dislocation, driven by a 2 Å “lift” of the Cα5 tail, a crucial step for dislodging the AHD of the G protein from the Ras-like domain. This AHD opening facilitates exposure of the canonical site 1, allowing for GTP binding and promoting free-nucleotide exchange and hydrolysis^37^. However, the precise location of site 2 en route from the S4-Gα_s_βγ to the S5-Gα_s_βγ state in the GPCR activation process, as well as its specific role in G protein signaling, remain unclear. Further investigation, potentially involving the structure of the S4 state in complex with full-length Gα_s_βγ, is required to address these questions.

Previous structural studies have shown that GPCR activation typically involves transitioning through multiple intermediate states^4–12^. From an energy landscape perspective, these transient intermediates represent high-energy substrates, making them challenging to characterize, especially when it comes to capturing transient GPCR-G protein complexes and studying their functions. Our study introduces a strategy to overcome these challenges by using 19F-qNMR to identify intermediate conformational states, trapping them with point mutations, and then structurally and functionally characterize them in complex with G proteins. While we used A_2A_R as a model system, it is clear that this ^19^F-qNMR-guided cryo-EM approach, where ^19^F-qNMR acts as a conformational indicator to guide the introduction of point mutations to bias GPCR towards an otherwise transient intermediate state. This strategy can be broadly applied to study transient complexes of other GPCRs and proteins during activation or inhibition. Deciphering the functions of these intermediate states and their complexes provides a more comprehensive understanding the complexity of GPCR signaling and opens therapeutic avenues by targeting specific disease-related conformations. As we advance our understanding of individual conformational states and their functions, it may become possible to design drugs based on their conformational selectivity, addressing signaling bias arising from conformational bias^6^.

## Methods

### Plasmid construction and transformation

The full-length human A_2A_R gene, originating from construct pPIC9K_ADORA2A, was generously provided by Prof. Takuya Kobayashi (Kyoto University, Kyoto, Japan). The C-terminally truncated construct A_2A_R_316, constructed in our previous study, has an integrated FLAG tag on the N-terminus and a poly-his tag on the C-terminus^7^. Based on this construct, the mutations V229C and R291A were described elsewhere^15^. All constructs were sequenced by a facility at Eurofins genomics, with the AOX1 primer pair of PF_AOX1_ and PR_AOX1_. Freshly prepared competent cells of strain *Pichia Pastoris* SMD 1163 (*Δhis4 Δpep4 Δprb1*, Invitrogen) were electro-transformed with *Pme*I-HF (New England Biolabs) linearized plasmids containing different mutant genes using a Gene Pulser II (Bio-Rad). High-copy clone selection was performed using an in-house protocol described previously^8,38^. A high-yield construct was then screened by an immunoblotting assay with both HRP-conjugated anti-FLAG (Bio-Teche, HAM85291) and HRP-conjugated anti-Poly-his (Bio-Techne, MAB050H), each diluted 1:2,000 in immunoblotting incubation buffer. Cell membranes were incubated with the antibodies at room temperature, followed by multiple washes with immunoblotting washing buffer^39^.

### Receptor expression, purification, and labeling

The screened WT* and mutants R291A, were pre-cultured on YPD [1% (w/v) yeast extract, 2% (w/v) peptone and 2% (w/v) glucose] plates containing 0.1 mg/mL G418. A single colony for each construct was inoculated into 4 mL YPD medium and cultured at 30 °C for 12 h, then transferred into 200 mL BMGY medium [1% (w/v) yeast extract, 2% (w/v) peptone, 1.34% (w/v) YNB (yeast nitrogen base) without amino acids, 0.00004% (w/v) biotin, 1% (w/v) glycerol, 0.1 M PB (phosphate buffer) at pH 6.5] and cultured at 30 °C for another 30 h. The cells were then transferred into 1 L of BMMY medium [1% (w/v) yeast extract, 2% (w/v) peptone, 1.34% (w/v) YNB without amino acids, 0.00004% (w/v) biotin, 0.5% (w/v) methanol, 0.1 M phosphate buffer at pH 6.5, 0.04% (w/v) histidine and 3% (v/v) DMSO, 10 mM theophylline] at 20 °C. 0.5% (v/v) methanol was added every 12 h. 60 h after induction by methanol, cells were harvested for purification.

The cell pellets were collected by centrifugation at 4000 × g for 20 min and washed one time with washing buffer (50 mM HEPES, 10% glycerol, pH 7.4) before the addition of breaking buffer (50 mM HEPES, pH 7.4, 100 mM NaCl, 2.5 mM EDTA, 10% glycerol) in a ratio of 4:1 (buffer: cells). The resuspended cell pellets were subject to disruption 3 times using a Microfluidizer at a pressure of 20,000 psi. Intact cells and cell debris were separated by low-speed centrifugation (8000 × g) for 30 min. The supernatant was collected and centrifuged at 100,000 × g for 2 h, and the precipitated cell membrane was then immediately dissolved in membrane lysis buffer (50 mM HEPES, pH 7.4, 100 mM NaCl, 0.5% LMNG-3 (Lauryl Maltose Neopentyl Glycol) and 0.1% CHS (cholesteryl hemisuccinate)) with rotation 2 h or overnight at 4 °C until the membrane was dissolved. Subsequently, Talon resin (Clontech) was added to the solubilized membranes and incubated for at least 2 h or overnight under gentle agitation.

The A_2A_R-bound Talon resin was washed twice with a buffer of 50 mM HEPES, pH 7.4, 100 mM NaCl, 0.02% LMNG-3 and 0.002% CHS and resuspended in the same buffer. The A_2A_R-bound Talon resin was then resuspended in buffer made of 50 mM HEPES, pH 7.4, 100 mM NaCl, 0.02% LMNG-3 and 0.002% CHS, and combined with 10–20 fold excess of the NMR label (2-bromo-*N*-(4-(trifluoromethyl)phenyl)acetamide, BTFMA, Apollo Scientific, Stockport, UK) under gentle agitation overnight at 4 °C. Another aliquot of NMR label was then added and incubated for an additional 6 h to ensure complete labeling. The A_2A_R-bound Talon resin was washed in a disposable column extensively with buffer containing 50 mM HEPES, pH 7.4, 100 mM NaCl, 0.02% LMNG-3 and 0.01% CHS, and apo A_2A_R was then eluted from the Talon resin with 50 mM HEPES, pH 7.4, 100 mM NaCl, 0.02% LMNG-3 and 0.01% CHS, 250 mM imidazole and concentrated to a volume of 5 mL. The XAC-agarose gel and A_2A_R were then incubated together for 2 h under gentle agitation. The functional A_2A_R was eluted with 50 mM HEPES, pH 7.4, 0.02% LMNG-3, 0.002% CHS, 100 mM NaCl, 20 mM theophylline. The eluted samples were concentrated to 1 mL by centrifugal filtration (MWCO, 3.5 KDa), and an extensive dialysis was performed to remove the theophylline in the sample. The functional apo A_2A_R was then prepared for NMR. All receptors described in this manuscript were purified using poly-his resin followed with a ligand-column, in which the A_2A_R antagonist xanthine amine congener (XAC) was conjugated to Affi-Gel 10 activated affinity media.

### Preparation of mini-Gα_s_β_γ_ heterotrimer

The plasmid for mini-Gα_s_399 was generously provided by Drs. Christopher G. Tate from MRC Laboratory of Molecular Biology, Cambridge and Javier García Nafría from Institute for Biocomputation and Physics of Complex Systems (BIFI) and Laboratorio de Microscopías Avanzadas (LMA), University of Zaragoza. It was expressed in the *E. coli* strain BL21(DE3). Cells were collected by centrifugation at 4,000 × g for 20 min and lysed by sonication. After another centrifugation, the supernatant was purified by Talon resin. The sample was loaded into a HiLoad16/60 column to obtain purified mini-Gα_s_ protein. The purified protein was concentrated to 3 mg/mL and flash frozen in liquid nitrogen and stored at -80 °C for further use. The expression and purification of the respective components and assembly to make the complex containing mini-Gα_s_β_1_γ_2_, and the preparation of nanobody Nb35, were all performed following the protocols described previously^1,40,41^.

### ^19^F NMR experiments

NMR samples typically consisted of 280–300 µL and were prepared in 50 mM HEPES buffer (pH 7.4) containing 100 mM NaCl, 0.02% LMNG-3, 0.002% CHS, with 30–50 µM of the protein sample, and 25 µM bendroflumethiazide as a chemical shift reference at −59.05 ppm. Samples were doped with 10% D_2_O and loaded into Shigemi tubes. In experiments involving ligand binding, 1 mM of ligand was added, and protein-ligand complexes were incubated for 20 min at room temperature prior to measurements.

All ^19^F NMR experiments were performed at 20 °C on a 600 MHz Varian Inova spectrometer equipped with a ^19^F dedicated resonance probe. Experimental parameters included a 16 µs 90° excitation pulse, an acquisition time of 200 ms, a spectral width of 15 kHz, and a repetition time of 1 s. Most spectra were acquired using 15,000–50,000 scans. Processing typically involved zero filling and exponential apodization equivalent to 15 Hz line broadening.

Peak assignments were performed by deconvolution using Lorentzian line fitting, optimizing intensity, and linewidth parameters. Exchange states were identified based on chemical shift perturbations observed across ligand-bound and apo conditions. For relaxation measurements, transverse relaxation times (T2) were obtained using a Carr-Purcell-Meiboom-Gill (CPMG) sequence with varying evolution times and fitted to a mono-exponential decay function^15^. Data analysis was performed with MestReNova 14.2 (Mestrelab Research), and relevant parameters, including linewidth and chemical shifts, are reported in the main text and Supplementary Information.

### GTPase hydrolysis assay

The GTPase hydrolysis assay was analyzed using a modified protocol of the GTPase-Glo^TM^ assay (Promega)^42^. The reaction was started by mixing 300 nM Gα_s_βγ with the purified receptors in varied concentrations with a final volume of 10 μL in the buffer containing 50 mM HEPES, pH 7.4, 100 mM NaCl, 0.002% CHS, 0.02% LMNG-3. For the GTP hydrolysis capacity of the S4 state as a function of ligand measurement, 5x and 25x ligand compared to receptor concentration was added. After 30 min incubation at room temperature, 10 μL 2xGTP-GAP solution containing 10 μM GTP, 1 mM DTT and the cognate GAP was added to each well, followed with a 120 min incubation at room temperature. For the Michaelis–Menten constant measurement, the 2xGTP-GAP solution containing 5–50 μM GTP was used. 20 μL reconstituted GTPase-Glo^TM^ reagent containing 5 μM ADP was added to each sample and incubated for another 30 min at room temperature with shaking. Luminescence was measured following the addition of 40 μL detection reagent and incubation for 10 min at room temperature using a BioTEK-Flx800 plate reader at 528±20 nm. The amount of GTP consumed was determined in a biochemical reaction by referencing a standard curve that relates light units (RLU) indicative of product formation to GTP concentration. The rate of the enzymatic reaction (velocity, v) was calculated by applying the Michaelis-Menten equation: v = V_max_/(1 + (K_m_/[S])). To facilitate the determination of *V*_*max*_ and *K*_*m*_, a Lineweaver-Burk plot was construct, which linearizes the relationship by graphing the reciprocal of the velocity (1/*v*) against the reciprocal of the substrate concentration (1/[*S*]). The calculations of initial rates were performed at 1.08 min in the linear reaction phase of catalysis. Analysis of data was performed by Excel and GraphPad Prism® 9.0.

### BODIPY-FL-GTP, BODIPY-FL-GDP binding, and nucleotide exchange

The nucleotide-binding assay utilized BODIPY-FL-GTP and BODIPY-FL-GDP, from Invitrogen™, each supplied as a mixture of two isomers with the fluorophore attached at either the 2′ or 3′ position on the ribose ring. BODIPY-FL-GTP could be hydrolyzed to BODIPY-FL-GDP. To form the GPCR-G protein complex, a solution containing 2 μM G protein and 100 μM receptor was incubated for 30 min at 22 °C. The fully activated state of the WT* receptor was achieved by supplementing the mixture with 10 mM NECA. Assays were conducted at 22 °C using 96-well half-area microtiter plates in a BioTek plate reader, with excitation at 475 nm and emission measured at 528 nm. The assay buffer comprised 20 mM HEPES (pH 7.4), 1 mM EDTA, and 10 mM MgCl_2_, supplemented with 0.01% LMNG-3 for protein stability. Initial kinetic data were acquired for 100 nM BODIPY- FL-GTP/BODIPY-FL-GDP in the absence of G protein for 70 seconds to establish a baseline fluorescence intensity. Subsequently, 200 nM heterotrimer G proteins, with or without the receptor, were added, and mixing was rapidly performed in the fluorescence cuvette. Data collection proceeded uninterrupted, and resulting kinetics spectra were plotted and fitted to a one-phase association function using GraphPad Prism 9.0. For the GTP-GDP exchange assay, the GPCR-G protein complex was formed as described, followed by incubation with 100 nM BODIPY-FL-GDP for 2 h. Baseline fluorescence intensity was measured for 70 seconds using the plate reader, after which 1 μM GTP (in a concentration of 10 × GDP), procured from Invitrogen™, was added to facilitate the exchange of BODIPY-FL-GDP to GTP. All experiments were repeated three times, and resulting kinetics spectra were analyzed using GraphPad Prism 9.0.

### GPCR-Gα_s_βγ complex digestion and Mass Spectrometry analysis

GPCR complex digestion was performed using sequencing-grade modified trypsin (Promega, V5113). Proteins were dissolved in 8 M urea and 50 mM ammonium bicarbonate, pH 7.8, containing 5 mM DTT and incubated at 37 °C for 1 h to reduce disulfide bonds. Following reduction, iodoacetamide was added to a final concentration of 15 mM to alkylate free thiol groups, and the reaction was incubated at room temperature for 30 min in the dark.

The denatured and reduced protein solution was diluted four-fold with 50 mM ammonium bicarbonate to lower the urea concentration to 1 M, optimizing conditions for enzymatic activity. Trypsin was added at an enzyme-to-protein ratio of 1:50 (w/w), and digestion was carried out overnight at 37 °C. To terminate enzymatic activity, formic acid was added to a final concentration of 1%. Standard samples containing 1 mM adenosine were prepared in P4 buffer. Peptide separation and analysis were performed using an Agilent 1260 Infinity LC system coupled to an Agilent 6120 Single Quadrupole Mass Spectrometer. A binary gradient system was used with solvent A (0.1% TFA in water) and solvent B (0.1% TFA in acetonitrile). A linear gradient was applied from 5% to 95% solvent B over 6 min, while solvent A decreased correspondingly from 95% to 5%.

### Gaussian accelerated molecular dynamics (GaMD)

GaMD is an enhanced sampling method that works by adding a harmonic boost potential to reduce the system energy barriers^43,44^. When the system potential ${\mbox{V}}({{\mbox{r}}}^{ \rightharpoonup })$ is lower than a reference energy E, the modified potential ${{\mbox{V}}}^{*}({{\mbox{r}}}^{ \rightharpoonup })$ of the system is calculated as:

1 $$\begin{array}{c}{V}^{*} ({{\rm{r}}}^{ \rightharpoonup })=V ({{\rm{r}}}^{ \rightharpoonup })+\Delta V ({{\rm{r}}}^{ \rightharpoonup })\\ \Delta V({{\rm{r}}}^{ \rightharpoonup })=\left\{\begin{array}{cc}\frac{1}{2}k { \left(E-V ({{\rm{r}}}^{ \rightharpoonup })\right)}^{2},& V({{\rm{r}}}^{ \rightharpoonup }) \, < \, E \\ 0,\hfill & V ({{\rm{r}}}^{ \rightharpoonup }) \, \ge \, E,\end{array}\right.\end{array}$$

where *k* is the harmonic force constant. The two adjustable parameters E and k are automatically determined on three enhanced sampling principles. First, for any two arbitrary potential values$\,{{\mbox{v}}}_{1}({{\mbox{r}}}^{ \rightharpoonup })$ and ${{\mbox{v}}}_{2}({{\mbox{r}}}^{ \rightharpoonup })$ found on the original energy surface, if ${{\mbox{V}}}_{1}({{\mbox{r}}}^{ \rightharpoonup }) < {{\mbox{V}}}_{2}({{\mbox{r}}}^{ \rightharpoonup })$, $\Delta V$ should be a monotonic function that does not change the relative order of the biased potential values; i.e., ${{\mbox{V}}}_{1}^{*}({{\mbox{r}}}^{ \rightharpoonup }) < {{\mbox{V}}}_{2}^{*}({{\mbox{r}}}^{ \rightharpoonup })$. Second, if ${{\mbox{V}}}_{1}({{\mbox{r}}}^{ \rightharpoonup }) < {{\mbox{V}}}_{2}({{\mbox{r}}}^{ \rightharpoonup })$, the potential difference observed on the smoothened energy surface should be smaller than that of the original; i.e., ${{\mbox{V}}}_{2}^{*}({{\mbox{r}}}^{ \rightharpoonup }){-{\mbox{V}}}_{1}^{*}({{\mbox{r}}}^{ \rightharpoonup }) < {{\mbox{V}}}_{2}({{\mbox{r}}}^{ \rightharpoonup }){-{\mbox{V}}}_{1}({{\mbox{r}}}^{ \rightharpoonup })$. By combining the first two criteria and plugging in the formula of ${V}^{*}({r}^{ \rightharpoonup })$ and$\,\Delta V$, we obtain

2 $${{\rm{V}}}_{\max }\le {{\rm{E}}} \le {{\rm{V}}}_{\min }+\frac{1}{{\rm{k}}},$$

Where ${{\mbox{V}}}_{\min }$ and ${{\mbox{V}}}_{\max }$ are the system minimum and maximum potential energies. To ensure that Eq. 2 is valid, *k* has to satisfy: ${\mbox{k}}\le 1/\left({{\mbox{V}}}_{\max }-{{\mbox{V}}}_{\min }\right)$. Let us define: ${\mbox{k}}={{\mbox{k}}}_{0}\cdot 1/\left({{\mbox{V}}}_{\max }-{{\mbox{V}}}_{\min }\right)$, then $0{ < {\mbox{k}}}_{0}\le 1$. Third, the standard deviation (SD) of $\Delta V$ needs to be small enough (i.e. narrow distribution) to ensure accurate reweighting using cumulant expansion to the second order: ${\sigma }_{\Delta V}=k(E-{V}_{{avg}}){\sigma }_{V}\le {\sigma }_{0}$, where ${V}_{{avg}}$ and ${\sigma }_{V}$ are the average and SD of $\Delta {\mbox{V}}$ with ${\sigma }_{0}$ as a user-specified upper limit (e.g., 10k_B_T) for accurate reweighting. When E is set to the lower bound $E={V}_{\max }$ according to Eq. 2, ${k}_{0}$ can be calculated as

3 $${{\rm{k}}}_{0}=\min \left(1.0,{{\rm{k}}}_{0}^{{\prime} }\right)=\min \left(1.0,\frac{{\sigma }_{0}}{{\sigma }_{{\rm{V}}}}\cdot \frac{{{\rm{V}}}_{\max }-{{\rm{V}}}_{\min }}{{{\rm{V}}}_{\max }-{{\rm{V}}}_{{\rm{avg}}}}\right),$$

Alternatively, when the threshold energy E is set to its upper bound ${\mbox{E}}={{\mbox{V}}}_{\min }+1/{\mbox{k}}$, ${{\mbox{k}}}_{0}$ is set to:

4 $${{\mathrm{k}}}_{0}={{\rm{k}}}_{0}^{{\prime\prime} }\equiv 1-\frac{{\sigma }_{0}}{{\sigma }_{{\rm{V}}}}\cdot \frac{{{\rm{V}}}_{\max }-{{\rm{V}}}_{\min }}{{{\rm{V}}}_{{\rm{avg}}}-{{\rm{V}}}_{\min }},$$

If ${{\rm{k}}}_{0}^{{\prime\prime} }$ is calculated between 0 and 1. Otherwise, k_0_ is calculated using Eq. 3.

### System setup and simulation analysis

The cryo-EM structure of wild-type NECA-bound A_2A_R bound by mini-Gα_s_ (PDB ID: 6GDG^18^) was used for setting up simulation systems of the wild-type NECA-bound cWT*-A_2A_R-mini-Gα_s_βγ, *apo* cR291A-mini-Gα_s_βγ, *apo* cWT*-A_2A_R-Gα_s_βγ and *apo* cR291A-Gα_s_βγ (Supplementary Table 2). The missing residues in the extracellular loop 2 (ECl2) and intracellular loop 3 (ICL3) of the receptor were modeled with Swiss-Modeller^45^. For the *apo* cR291A-mini-Gα_s_βγ, the simulation structure was generated by mutanting the correspoding R291A and V229C in the wild-type system and deleting the agonist NECA. To build the wild-type *apo* A_2A_R bound by the full-length of Gs protein (*apo* cWT*-A_2A_R-Gα_s_βγ), the Swiss-Modeller^45^ was used to build the Gs protein with a geometry and orientation similar to the Gs protein in the fully active state of the β_2_AR-Gs complex (PDB ID: 3SN6^1^). The coordinates of the GDP and Mg^2+^ were obtained by aligning the Ras domain of the crystal structure of Gs-bound GDP (PDB ID: 6AU6^46^) to the modeled full-length Gs protein bound by the A_2A_R.The simulation structure of the *apo* cR291A-Gα_s_βγ was generated by substituting residues Arg291 and Val232 in the wild-type system (*apo* cWT*-A_2A_R-Gα_s_βγ) with Ala and Cys, respectively.

VMD was used to insert the NECA-bound cWT*-A_2A_R-mini-Gα_s_βγ, *apo* cR291A-mini-Gα_s_βγ, *apo* cWT*-A_2A_R-Gα_s_βγ, and *apo* cR291A-Gα_s_βγ complex into POPC (palmitoyl-2-oleoyl-sn-glycero-3-phosphocholine) lipids to prepare simulation systems. In each simulation system, the protein and lipid bilayer were solvated with TIP3P water molecules in a box of 11.2 nm x 13.1 nm x 14.6 nm with the periodic boundary condition. The system charge was neutralized with 150 mM NaCl. The CHARMM36m parameter set^47–49^ was used for the proteins and lipids, and Guanosine diphosphate (GDP). Force field parameters of the NECA agonist were obtained from the ParamChem web server^50^. The four simulation systems were first energy minimized for 5,000 steps with constraints on the heavy atoms of the proteins and phosphor atom of the lipids. The hydrogen-heavy atom bonds were constrained using the SHAKE algorithm and the simulation time step was set to 2.0 fs. The particle mesh Ewald (PME) method^51^ was employed to compute the long-range electrostatic interactions and a cutoff value of 9.0 Å was applied to treat the non-bonded atomic interactions. The temperature was controlled using the Langevin thermostat with a collision frequency of 1.0 ps^−1^. The system was then equilibrated using the constant number, volume, and temperature (NVT) ensemble at 310 K for 250 ps and under the constant number, pressure, and temperature (NPT) ensemble at 310 K and 1 bar for another 1 ns with constraints on the heavy atoms of the protein, followed by 10 ns short conventional MD (cMD) without any constraint.

The GaMD module implemented in the GPU version of AMBER22^52–54^ was then applied to perform the simulations of NECA-bound cWT*-A_2A_R-mini-Gα_s_βγ, *apo* cR291A-mini-Gα_s_βγ, *apo* cWT*-A_2A_R-Gα_s_βγ, and *apo* cR291A-Gα_s_βγ. GaMD simulations included an 8 ns short cMD run used to collect the potential statistics for calculating GaMD acceleration parameters, a 56 ns GaMD equilibration after adding the boost potential, and finally three independent 2,000 ns GaMD production simulations with randomized initial atomic velocities for the systems of the NECA-bound cWT*-A_2A_R-mini-Gα_s_βγ and *apo* cR291A-A_2A_R-mini-Gα_s_βγ complex, and 3,000 ns GaMD production simulations for the systems of *apo* cWT*-A_2A_R-Gα_s_βγ and *apo* cR291A-Gα_s_βγ with randomized initial atomic velocities. The average and SD of the system potential energies were calculated every 800,000 steps (1.6 ns). All GaMD simulations were performed at the “dual-boost” level by setting the reference energy to the lower bound. One boost potential was applied to the dihedral energetic term and the other to the total potential energetic term. The upper limit of the boost potential SD, σ_0_ was set to 6.0 kcal/mol for both the dihedral and the total potential energetic terms.

For each simulation system, all three GaMD production trajectories were combined together for analysis with CPPTRAJ^55^. The distance between the NPxxY motif of the receptor and the last five residues of Gα_s_ α5 helix (A_2A_AR:NPxxY-Gα_s_:α5 distance), the distance between the receptor TM3 and TM6 intracellular ends (measured by the distance between the Cα atoms of receptor residues Arg102^3.50^ and Glu228^6.30^), the root-mean-square derivation of GDP (GDP RMSD) relative to the simulation starting structure were selected as reaction coordinates. The angle between the Ras domain and α helical domain (AHD) was used as another reaction coordinate to indicate their relative orientation, which was defined by the two vectors of Gα_s_ AHD and Gα_s_ Ras domain. Vector 1 went through the Gα_s_ AHD and A161 centers, and vector 2 went through the Gα_s_ Ras domain and E299 centers. The PyReweighting^56^ toolkit was applied to reweight GaMD simulations to recover the original free energy profiles of the simulation systems. 2D free energy profiles were computed using the combined trajectories from all the three independent GaMD simulations for each system with the A_2A_R:NPxxY-Gα_s_:α5 distance, TM3-TM6 distance, GDP RMSD and the angle between the Ras domain and AHD as reaction coordinates. A bin size of 1.0 Å was used for the A_2A_R:NPxxY-Gα_s_:α5 distance, TM3-TM6 distance, and GDP RMSD. A bin size of 6.0° was used for the angle between the Ras domain and AHD as the reaction coordinate. The cutoff was set to 500 frames for 2D free energy calculations.

### Preparation of the A_2A_R-mini-Gα_S_β_1_γ_2_-Nb35 complex

A_2A_R, mini-Gα_S_-β_1_γ_2,_ and Nb35 were mixed in a molar ratio of 1:2:4 to yield a final complex concentration of 1 mg/mL. To this mixture, 0.1 U of apyrase was added, followed by an overnight incubation at 4 °C. The mixture was then concentrated with a 100 kDa MWCO Amicon filter and injected onto a Superdex200 Increase 10/300 GL gel filtration column equilibrated with buffer (50 mM HEPES, 100 mM NaCl, 0.002% LMNG-3, 0.0002% CHS (w/v)). Monodisperse fractions were concentrated with a 100 kDa MWCO Amicon filter immediately prior to cryo-EM grid preparation.

Negative staining of the complex was performed with 0.75% uranium formate, following an established protocol^57^. Grids were examined using an FEI T12 microscope operated at 120 kV, and images were recorded using a 4 K x 4 K charge-coupled device (CCD) camera (UltraScan 4000, Gatan).

### Cryo-EM sample preparation and data acquisition

Freshly prepared A_2A_R-mini-Gα_S_-β_1_γ_2_-Nb35 complex at a final concentration of 1 mg/mL, was applied to glow-discharged gold grids coated with either holey carbon film (Quantifoil, 300 mesh 1.2/1.3, Au) or holey gold film (UltrAuFoil, 300, mesh 1.2/1.3). These grids were then plunge-frozen using a Vitrobot Mark IV with a blotting time of 4 s and blotting force of 0, at 4 °C and 100% humidity. Grids were subsequently examined and screened using an FEI Tecnai Arctica operated at 200 kV and equipped with an XFEG and a Gatan K3 camera. Cryo-EM data collection was performed on a Titan Krios at the UCSF Cryo-EM Center for Structural Biology, operated at an acceleration voltage of 300 kV, equipped with an XFEG, a BioQuantum energy filter (slit width set to 20 eV) and a K3 camera (Gatan).

All cryo-EM datasets were collected using SerialEM^58^. A multishot collection (3 × 3 arrays) was employed, incorporating beam-tilt compensation and a maximum image shift of 3.5 micros. All images were acquired with a nominal magnification of 105 K, resulting in a super-resolution pixel size of 0.4175 Å (physical pixel size of 0.835 Å). The defocus range was set from -0.8 μm – -1.8 μm. A total of 8,805 images were collected, each was dose-fractionated into 80 movie frames with a total exposure time of 2.024 s, resulting in a total fluence of ~47.7 electrons per Å^2^. Data collection statistics are shown in Supplementary Table 1.

### Image process

A total of 8,805 movie stacks were motion-corrected, dose-weighted, and binned by Fourier cropping to the physical pixel size of 0.835 Å on-the-fly using MotionCor2^59^. Motion-corrected, does-weighted sums were used for contrast transfer function (CTF) determination and resolution estimation in cryoSPARC^60^. 424,536 particles were picked from randomly selected 500 micrographs using a cryoSPARC blob picker with a diameter of 80-150 Å. These particles were subjected to ab-initio reconstruction and multi-round heterogeneous refinement. One class with distinct features of A_2A_R bound to mini-Gα_s_β_1_γ_2_-Nb35 was identified, from which 16 different projection images were created for template picking, yielding 7,282,317 particles from all micrographs.

After removing junk particles by extensive 2D classifications, 2,328,810 particles were selected for ab-initio reconstruction and multi-round heterogenous refinement. One distinct class of 307,568 particles was identified for further non-uniform refinement of the A_2A_R-mini-Gα_s_β_1_γ_2_-Nb35 complex, yielding a reconstruction with a global resolution of 3.04 Å. This particle stack was then exported to RELION^61^ for multiple rounds of 3D refinement (initial low-pass filter: 10 Å; mask diameter: 360 Å; reference mask: no) to produce a new reconstruction, from which a mask without the detergent micelle was generated by using the segment map function in Chimera^62^ (initial threshold: 0.0001; extend_inimask: 4; width_soft_edge: 4). Particle subtraction function in RELION was applied by using this mask to generate a micelle removed particle stack.

Next, we applied 3D classification to the micelle subtracted particles with a reference model without micelle (reference mask: generated last step; initial low-pass filter: 10 Å; mask diameter: 260 Å; regularization parameter T: 3; number of iterations: 50; number of classes: 5; perform local angular searches: no). Each 3D class was further refined in RELION (initial low-pass filter: 3.2 Å; mask diameter: 260 Å; angular sampling interval: 1.8°; local search from auto-sampling: 0.9°). Final 3D reconstructions were calculated in cisTEM^63^ with default settings without further refinement. Of the five classes, one class contained 71,547 particles in the S4 state, and one class with 65,016 particles was identified as S4_d2_. The remaining 171,005 particles, although aligning well with the S4 state in terms of C-α displacement, did not yield high resolution after multi-rounds of 3D classification and refinement in RELION and were therefore discarded from the final reconstruction.

We performed a 3D variability analysis (3DVA) in cryoSPARC on the full stack of 307,568 particles to reveal G-protein motion, with or without applying a mask to the A_2A_R receptor. From each volume series (20 frames), five frames with slightly different conformations were manually identified. These frames were imported as five separate classes in 3D classification with identical settings, then refined in RELION. Four classes aligned with the S4 state, while one distinct class was identified as S4_d1._ A final 3D reconstruction was calculated in cisTEM^63^ with default settings. Following independent 3DVA, a small percentage of particles overlapped with the S4 and S4_d2_ conformations and were excluded, resulting in 64,031 particles for S4, 43,472 for S4_d1_, and 46,863 for S4_d2_. The numeric resolution was determined from Fourier Shell Correction (FSC) using the criterion of FSC = 0.143^64^. The final map was sharpened by a B factor of -10 Å^2^ in cisTEM and used for model building and figure generation.

### Model building

For model building, the initial models were generated by fitting the existing coordinates of the activated state of the A_2A_R-mini-Gα_S_-β_1_γ_2_-Nb35 complex (PDB: 6GDG) into our cryo-EM density maps using ChimeraX^65^. Discrepancies between the initial models and the density maps were then manually built and refined in ISOLDE^66^ and coot^67^. Subsequent refinements were performed in Phenix^68^ with secondary structure constraints. The models were validated by wwPDB validation server^69^ and no major issues were reported. A summary of the parameters used in data collection and model building is provided in Supplementary Table 1.

### Data representation

All statistical tests such as GTP hydrolysis assessments were conducted using GraphPad Prism 9.0. The central point of all data points gives the mean value with s.d. for all data unless otherwise specified. The atomic models (Figures and Movies) were visualized using UCSF ChimeraX and PyMoL (The PyMOL Molecular Graphics System, Version 2.0 Schrödinger, LLC.). In the model comparison, the target models were color-coded based on the C-α displacement of each residue relative to the reference S4 state.

### Reporting summary

Further information on research design is available in the Nature Portfolio Reporting Summary linked to this article.

## Supplementary information

Supplementary Information

Description of Additional Supplementary Files

Supplementary Movie 1

Supplementary Movie 2

Supplementary Movie 3

Supplementary Movie 4

Reporting Summary

Transparent Peer Review file

## Data Availability

The atomic coordinates of the intermediate A_2A_R-mini-Gα_s_βγ structures solved in this study have been deposited in the PDB under accession codes 9EE8, 9EE9, and 9EEA. The corresponding cryo-EM maps have been deposited in the EMDB under accession codes EMD-47951, EMD-47952, and EMD-47953. Additional structural data used in this study are available under accession codes 3SN6, 6AU6, 6EG8, 6GDG, and 7ARO. Input, output, and parameters files for NECA bound cWT*-A2AR-mini-Gαsβγ and apo cR291A-mini-Gαsβγ are available on Figshare (10.6084/m9.figshare.28079807.v1; 10.6084/m9.figshare.28079783.v1). Additional simulation files, including trajectories, can be obtained by contacting the co-corresponding author Yinglong Miao (Yinglong_Miao@med.unc.edu). Additional Source Data are available on Figshare through the following link: 10.6084/m9.figshare.28233065.v2.
