# Supplementary material for: Structure and function of a near fully-activated intermediate GPCR-Gαβγ complex
Source: Nat Commun. 2025 Jan 28;16:1100. doi: 10.1038/s41467-025-56434-4 (PMC11775185; doi:10.1038/s41467-025-56434-4)
Supplement: Supplementary file 1 — Supplementary Information [file 41467_2025_56434_MOESM1_ESM.pdf]

Supplementary Figures

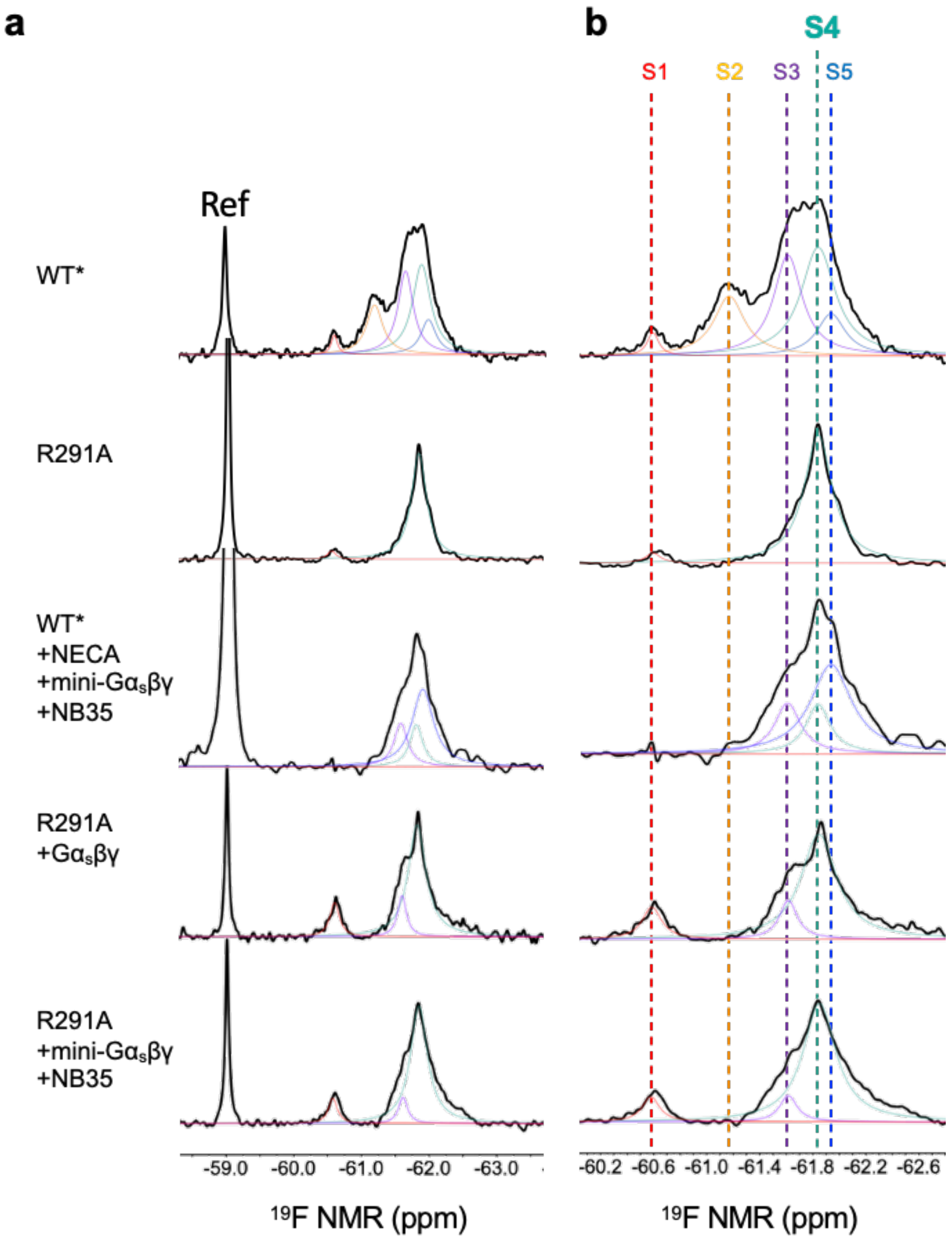

Supplementary Figure 1: Conformational profiles of the mutant R291A and its complexes probed by ^{19}F-qNMR. (A) and **(B),** Deconvoluted conformational profiles probed by ^{19}F-qNMR as a function of mini-G$\alpha_s\beta\gamma$ and G$\alpha_s\beta\gamma$ (S4-G$\alpha\beta\gamma$), in reference to the WT*-mini-G$\alpha_s\beta\gamma$+NB35 (S5-G$\alpha_s\beta\gamma$), representing the conformational profile for the structure PDB ID: 6GDG.

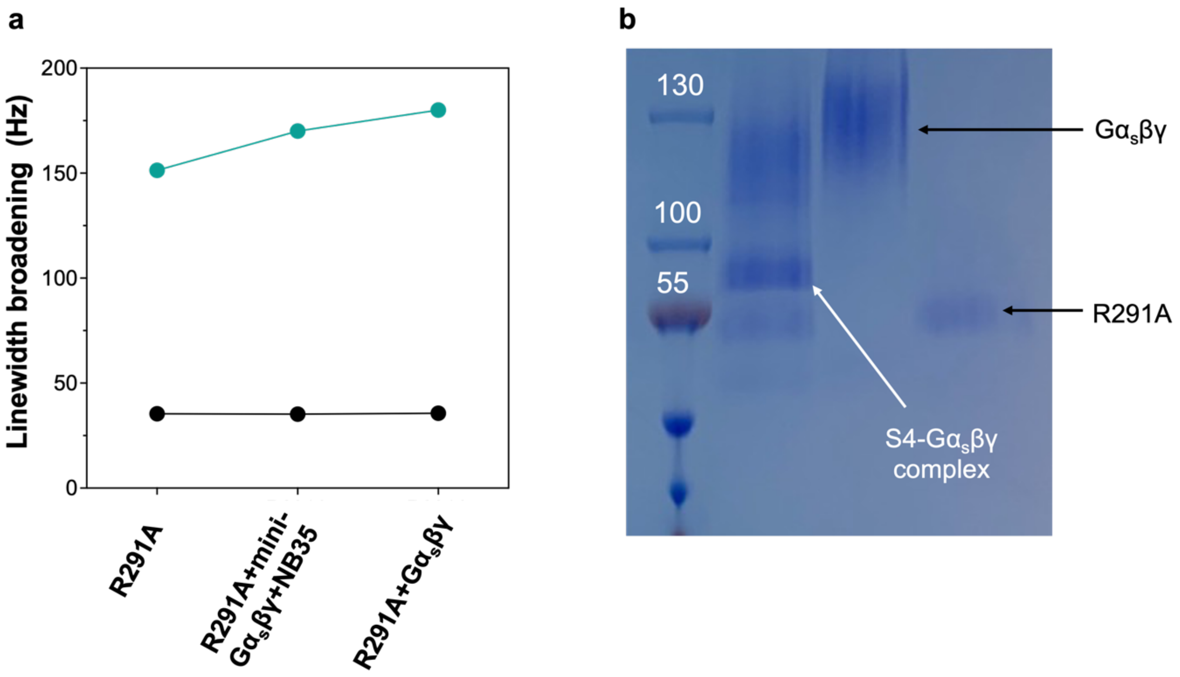

Supplementary Figure 2: Both linewidth broadening of ^{19}F-qNMR resonance for S4 intermediate and native-PAGE indicated the complex formation between the S4 and G protein. a, Linewidths of the S4 resonance for R291A (black), R291A-Gα$_s$βγ (teal), and R291A-mini-Gα$_s$βγ-NB35 (teal), demonstrating the increase in linewidths corresponding to complex formation. **b,** Native-PAGE for R291A and Gα$_s$βγ interaction.

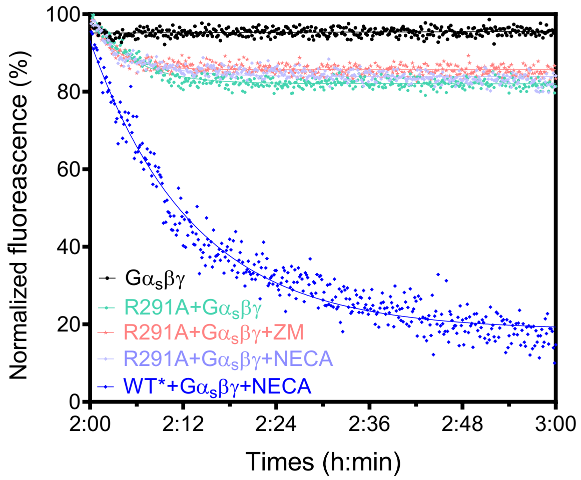

Supplementary Figure 3: GTP-GDP exchange rates of R291A mutant and WT*. R291A mutant without ligand (green), with ZM241385 (red), with NECA (cyan), and WT receptor with NECA (blue) are shown. R291A exhibits slower exchange rates, especially with ZM241385, while WT with NECA demonstrates the fastest rate, indicating a fully active state.

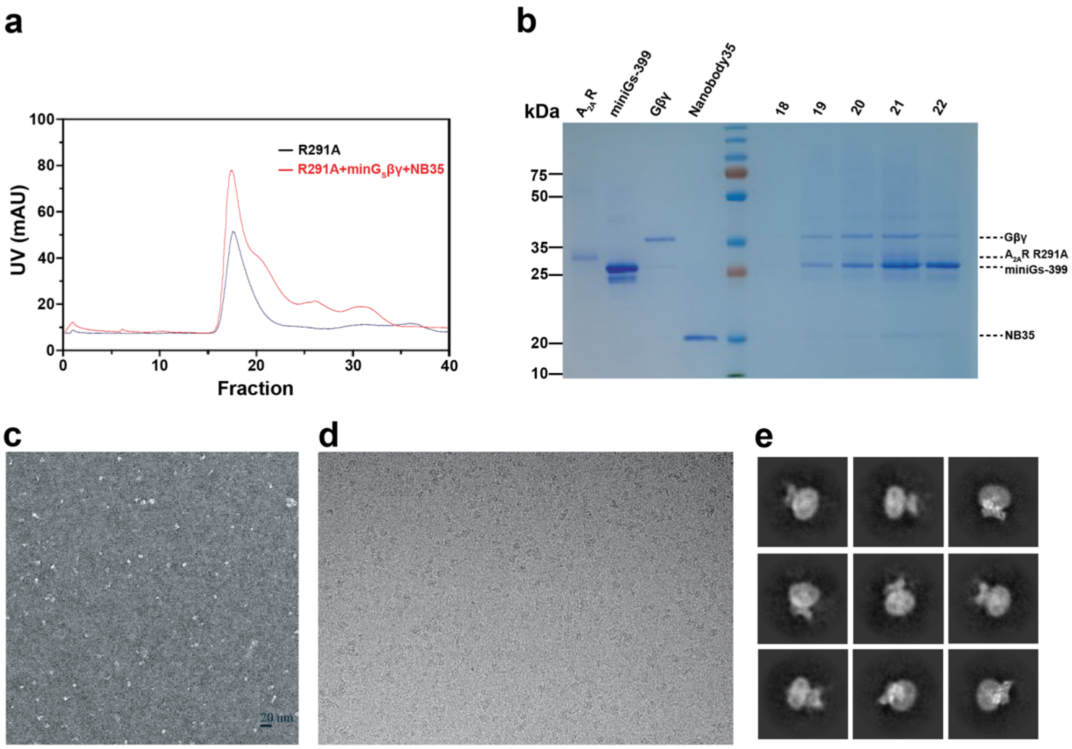

Supplementary Figure 4: Preparation of $A_{2A}R$ sample and cryo-EM screening. a, Size exclusion chromatography of purified $A_{2A}R$-R291A with and without mini-$G_s\beta\gamma$+NB35. **b,** SDS-PAGE of the indicated fraction, corresponding to R291A+mini-$G_s\beta\gamma$+NB35. **c**, Negative staining of the R291A+ mini-$G\alpha_s\beta\gamma$+NB35 complex. **d**, Representative cryo-EM micrograph of the complex. **e**, Representative 2D average image of the complex.

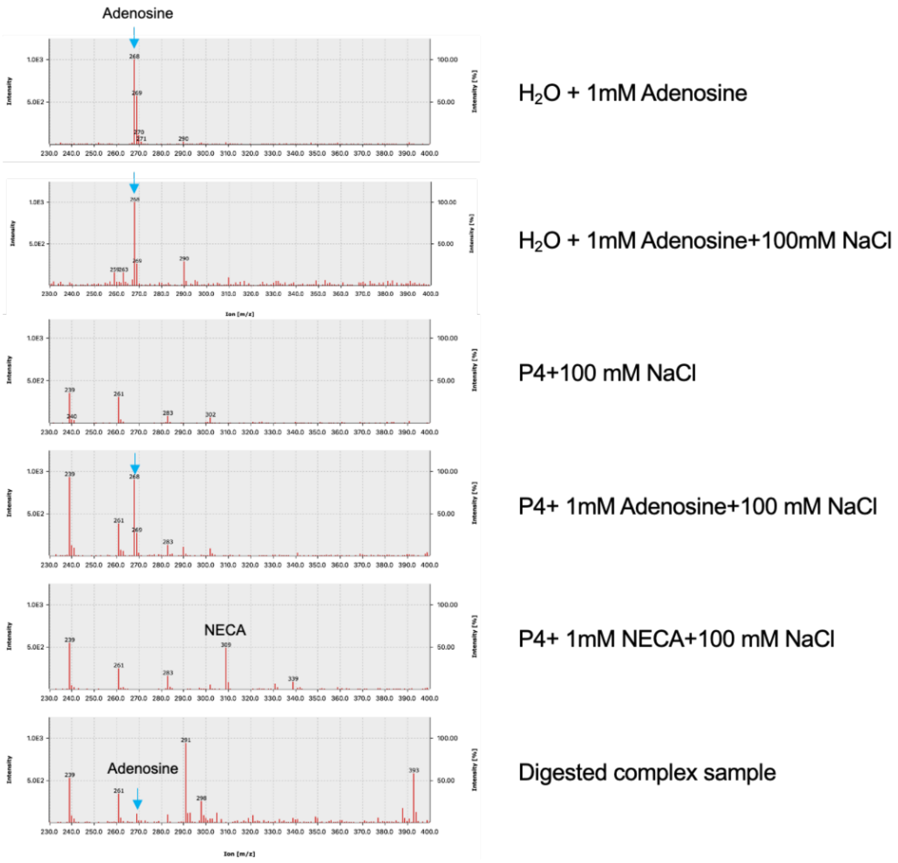

Supplementary Figure 5. Mass spectrometry (MS) analysis of samples containing adenosine, NECA, and adenosine with sodium adducts. The spectra display the mass-to-charge (m/z) ratios of detected ions, with the x-axis representing m/z and the y-axis showing relative intensity. Key peaks corresponding to adenosine (m/z = X) and NECA (m/z = Z) are annotated in the respective spectra. The presence of these specific ions confirms the identity of the sample components.

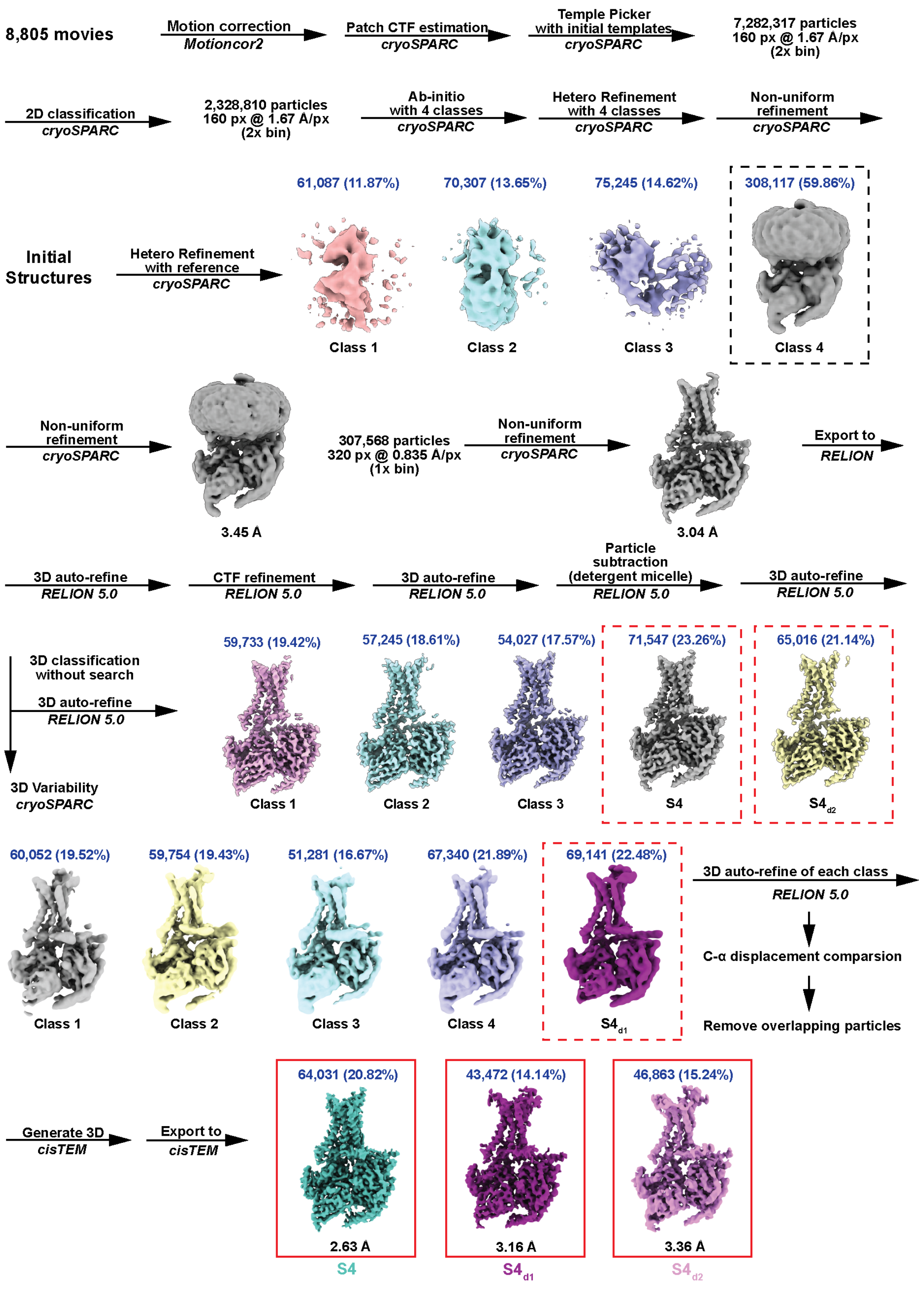

Supplementary Figure 6: Cryo-EM data and image processing workflow. Schematic flowchart illustrating the image processing approach for A_{2A}R-R291A. Thumbnail images of each 3D class or refinement are presented, with particle counts indicated in blue. Red dashed boxes highlight selected 3D classes. The final Cryo-EM maps, represented by red boxes, depict the $S4_{d1}$, $S4_{d2}$, and S4 conformations.

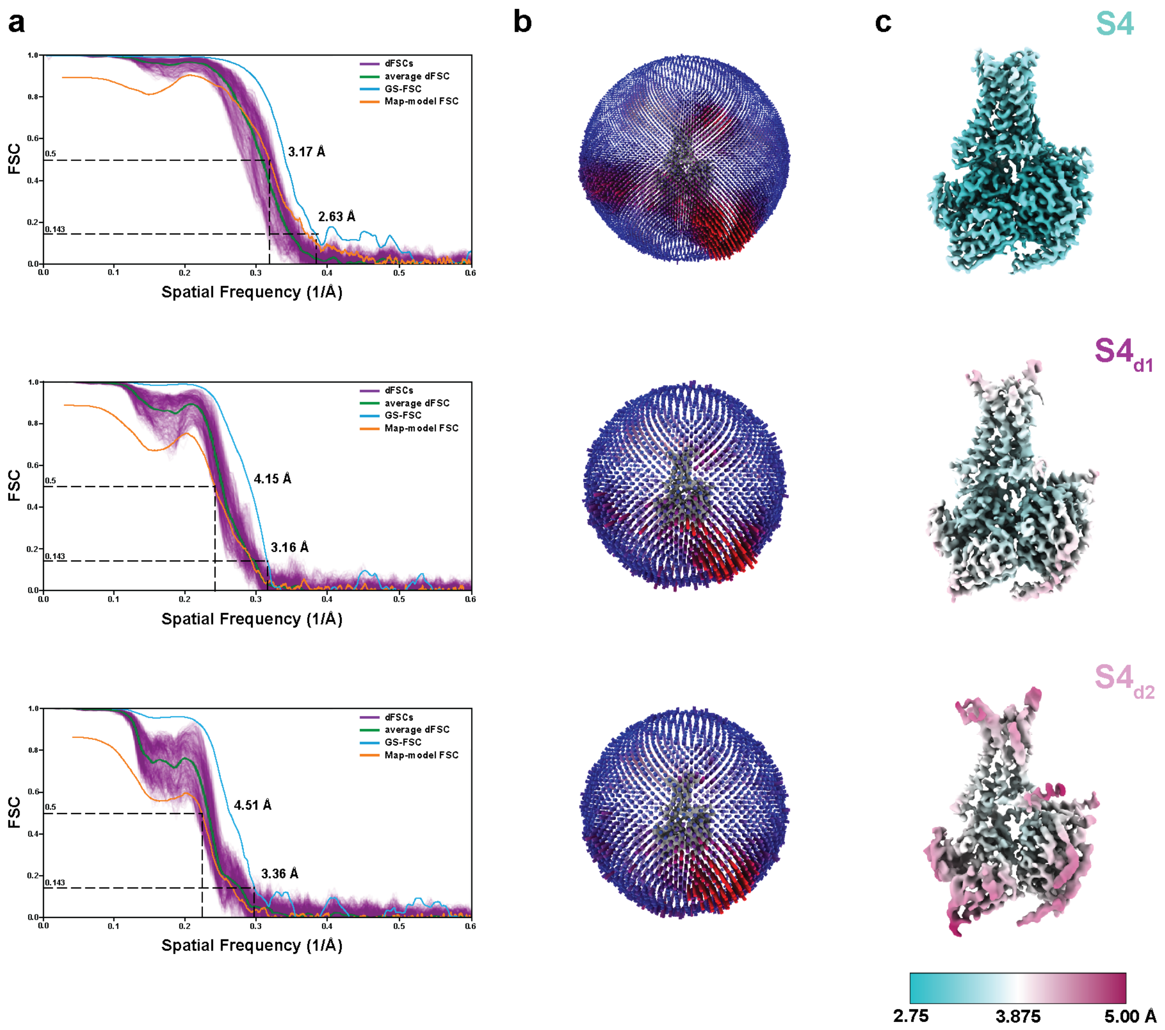

Supplementary Figure 7: Cryo-EM density and atomic model quality. a, Fourier shell correlation (FSC) curves, including directional FSCs (dFSCs), the average dFSC, the 'gold-standard' FSC, and the model-to-map FSC. **b,** Histogram representation of the Euler angle distribution of the final particles used in the reconstruction. **c,** Cryo-EM map colored by local resolution estimates, calculated using RELION, with a resolution scale bar ranging from 2.75 to 5 Å

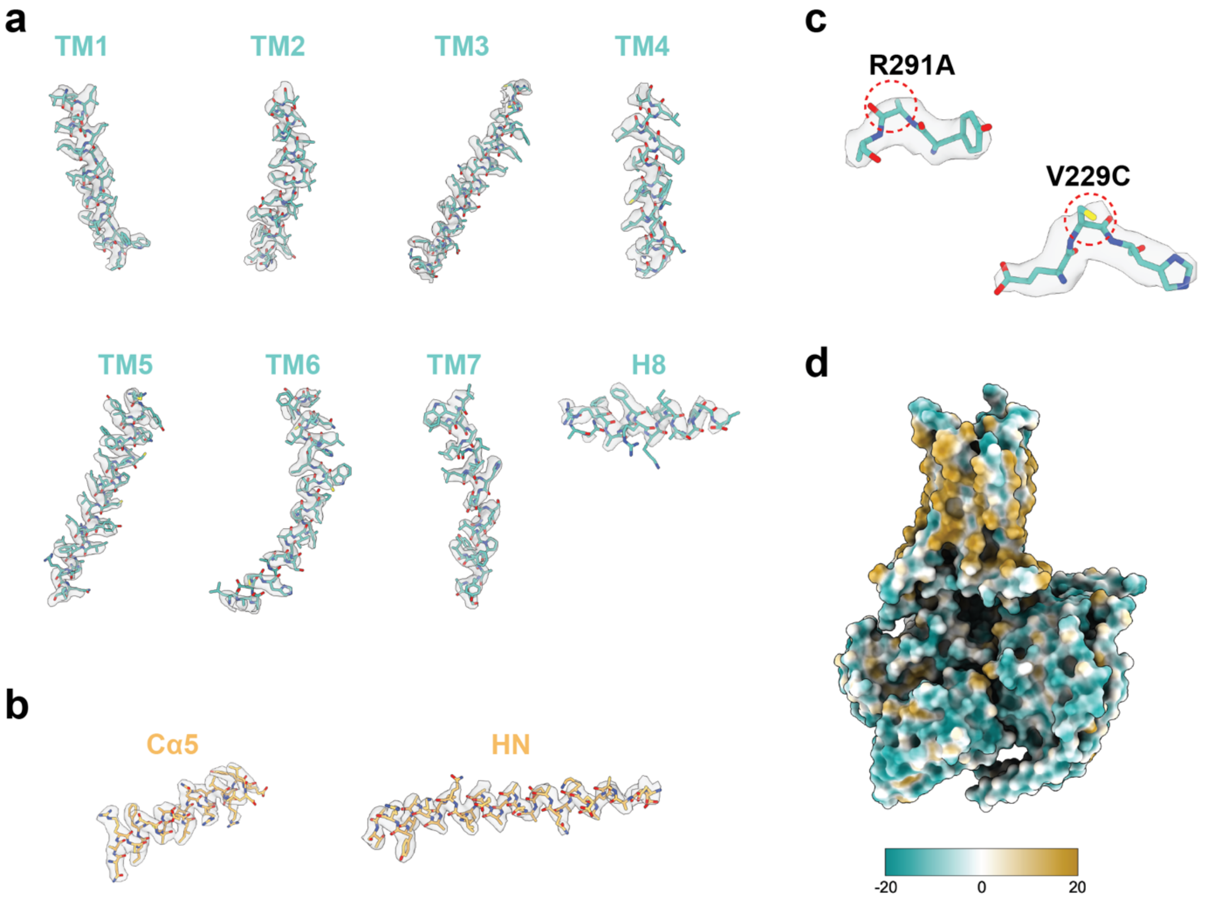

Supplementary Figure 8: Representative cryo-EM densities from selected structural features of the S4 state. **a**, Densities of all transmembrane helices. **b**, Representative densities of mini-G. **c,** Cryo-EM densities of R291A and V229C. **d,** Representation of surface hydrophobicity.

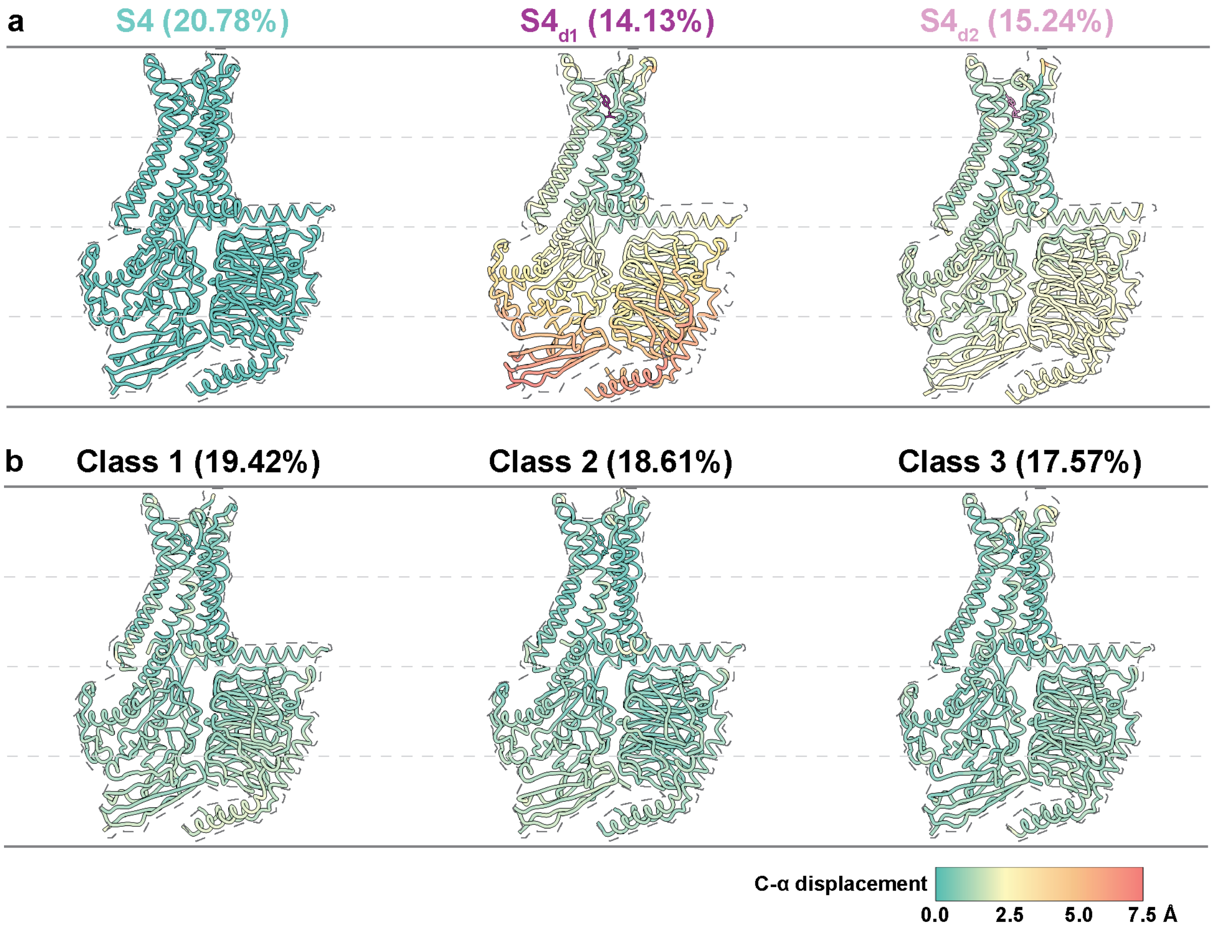

Supplementary Figure 9: C-α displacement analysis of S4 dynamic snapshots. a, C-α displacement representation of the S4, $S4_{d1}$, $S4_{d2}$ states, with the S4 state outlined in dashed lines for reference. **b,** Class 1-3 represent particles excluded from the final high-resolution reconstruction after 3D auto-refinement in RELION. These classes show strong structural similarities to the S4 state, as indicated by the dashed outline for comparison. C-α displacement calculations for these classes illustrate conformational similarities. The C-α displacement color bar, mapped to each C-α atom, ranges from 0.0 Å (teal) to 7.5 Å (red), with intermediate values at 2.5 Å (light yellow) and 5.0 Å (peach).

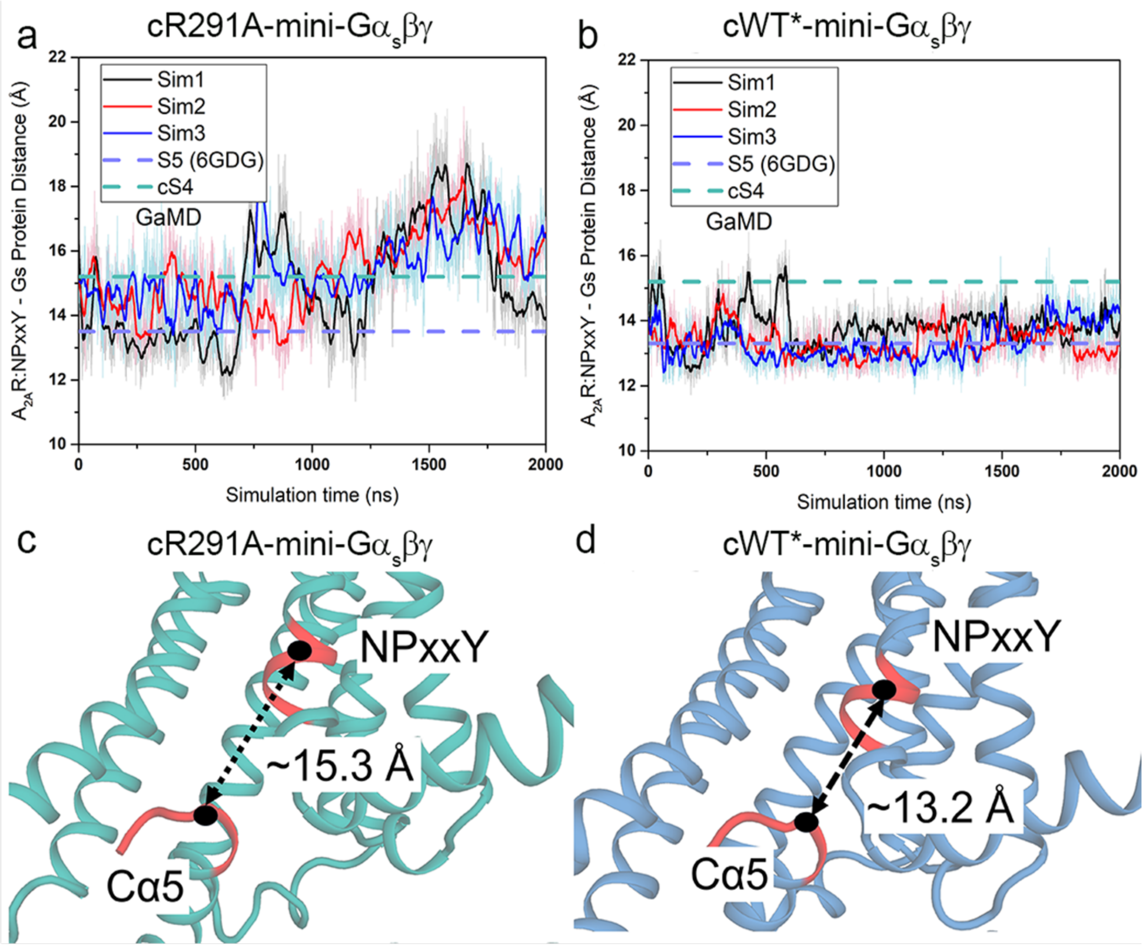

Supplementary Figure 10: GaMD simulations for cR291A-mini-Gα$_s$βγ and cWT*-mini-Gα$_s$βγ. **a-b**, Time course of the center-of-mass (COM) distance between the receptor NPxxY motif in TM7 and the Cα5 helix (A_{2A}R:NPxxY-Gαs:Cα5 distance) calculated from the GaMD simulations of cR291A-mini-Gα$_s$βγ (**a**) and cWT*-mini-Gα$_s$βγ (**b**) complex systems. **c-d**, Representative low-energy conformations of the cR291A-mini-Gα$_s$βγ (**c**) and **(d)** cWT*-mini-Gα$_s$βγ complex identified from the GaMD simulations. The receptor NPxxY motif and the last five residues of Gα5 helix were colored in orange.

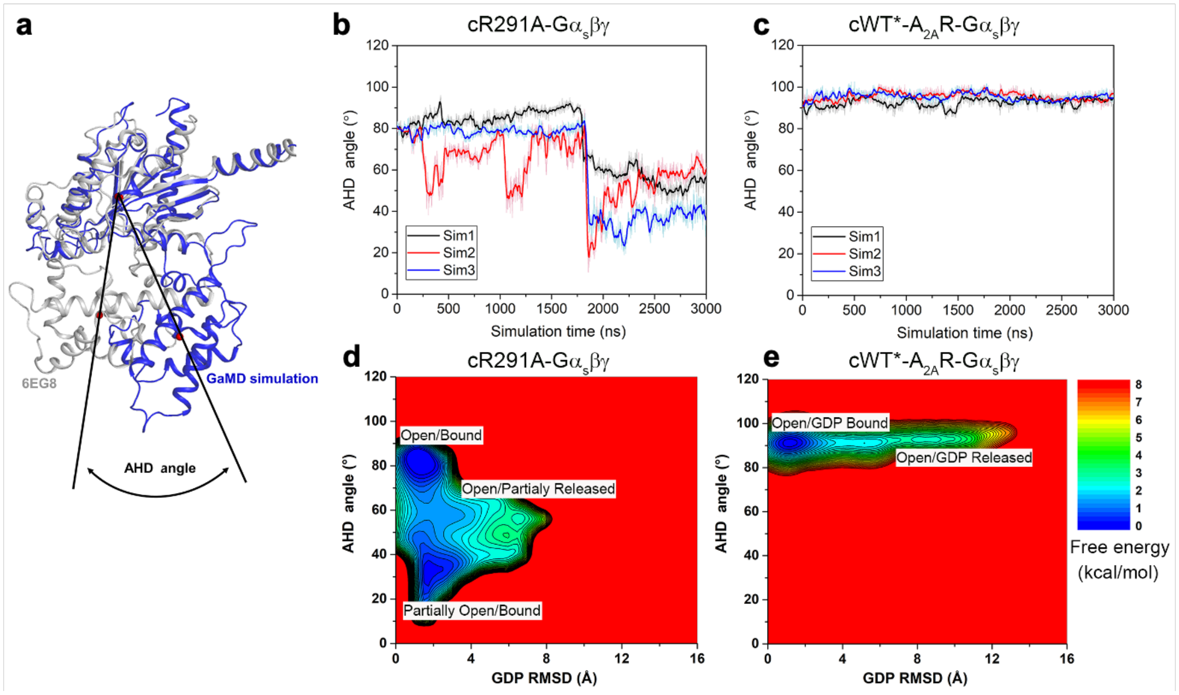

Supplementary Figure 11: The behaviors of AHD in cS4 and cS5 mediated G protein. a, The AHD angle is illustrated by comparing the GaMD simulation conformation with the inactive conformation of Gα_s (PDB: 6EG8); Vector 1 goes through centers of the Gα_s AHD and Ras-like domain of the inactive conformation (6EG8). Vector 2 goes through the centers of the Gα_s AHD and Ras-like domain of the GaMD simulation conformations after aligning the Ras-like domain to the inactive conformation of Gα_s. **b-c,** The time course of the AHD angle calculated from the GaMD simulations for **b,** *apo* cR291A-G$\alpha_s\beta\gamma$, and **c,** *apo* cWT*-A$_{2A}$R-G$\alpha_s\beta\gamma$ complexes. **d-e,** 2D free energy profiles of **d,** *apo* cR291A-G$\alpha_s\beta\gamma$, and **e,** *apo* cWT*-G$\alpha_s\beta\gamma$ complexes calculated from GaMD simulations regarding the GDP RMSD and the AHD angle.

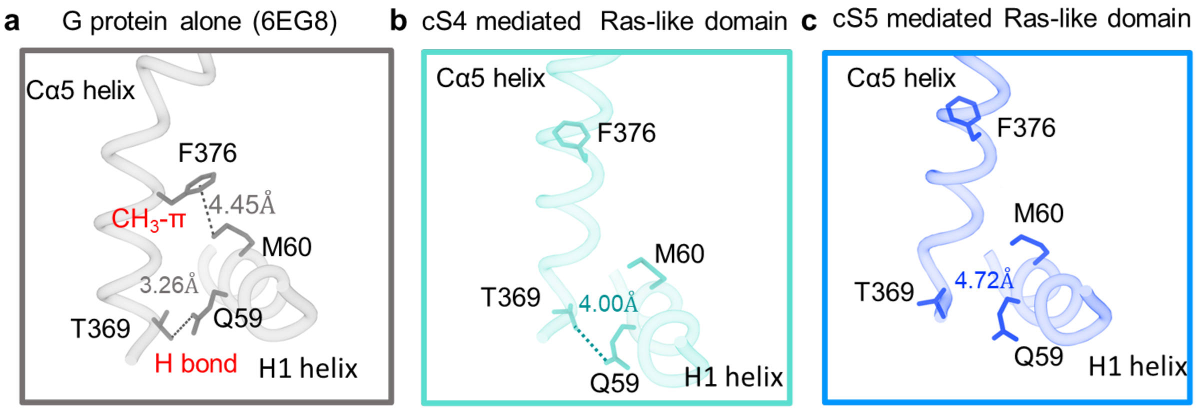

Supplementary Figure 12: The relative positions of Cα5 and H1 helices. a, G protein is inactive (6EG8). **b,** partially activated (cS4 mediated). **c,** fully activated (cS5 mediated).

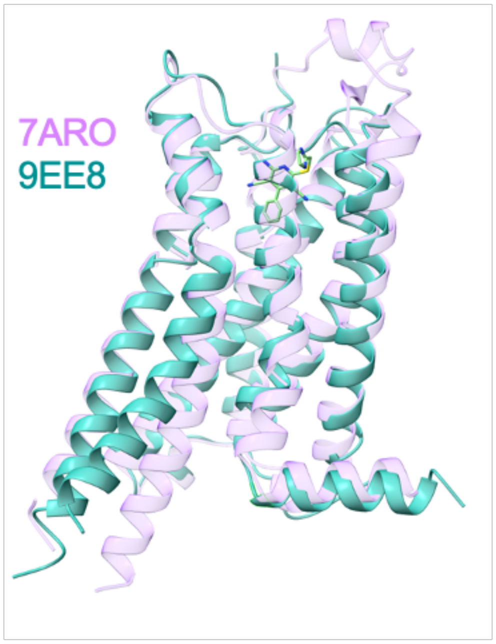

Supplementary Figure 13: Comparison of two $A_{2A}R$ structures. S4 state structure (PDB ID: 9EE8) from $A_{2A}R$-R291A mutant (teal) in our structure and crystal structure (PDB ID: 7ARO) of $A_{2A}R$ bound to the non-ribose partial agonist LUF5833 (light purple).

Supplementary Table 1 Cryo-EM data collection, refinement, and validation statistics

	A_{2A}R-R291A '**S4**' EMDB-47951 PDB 9EE8	'**S4**$_{d1}$' EMDB-47952 PDB 9EE9	'**S4**$_{d2}$' EMDB-47953 PDB 9EEA
**Data collection and processing**			
Magnification		EFTEM 105,000	
Voltage (kV)		300	
Electron exposure (e^-/Å^2)		47.7	
Defocus range (μM)		-0.8-1.8	
Pixel size (Å)		0.835	
Symmetry imposed		C1	
Initial particle images/refined (no.)		7,282,317/307,568	
Final particle images (no.)	64,031	43,472	46,863
Map resolution (Å)	2.63	3.16	3.36
FSC threshold	0.143	0.143	0.143
Map resolution range (Å)			
**Refinement**			
Initial model used (PDB code)	6GDG	6GDG	6GDG
Model resolution (Å)	3.17	4.15	4.51
FSC threshold	0.5	0.5	0.5
Model Resolution range (Å)			
Map sharpening *B* factor (Å^2)	-10	-10	-10
Model composition			
Non-hydrogen atoms	14,843	14,757	14,659
Protein residues	1,025	1,020	1,011
Ligands	ADN:1	ADN:1	ADN:1
B factor (Å^2)			
Protein (min/max/mean)	28/167/86	73/213/131	49/170/94
Ligands	101/110/106	139/147/144	79/97/89
R.m.s. deviations			
Bond lengths (Å)	0.004	0.002	0.003
Bond angles (°)	0.604	0.558	0.580
Validation			
MolProbity score	1.33	1.87	2.02
Clash score	4.17	10.62	11.51
Poor rotamers (%)	0.26	0.00	0.40
Ramachandran plot			
Favored (%)	97.32	95.32	93.17
Allowed (%)	2.68	4.58	6.83
Outliers (%)	0.00	0.10	0.00

Supplementary Table 2 Summary of GaMD Simulations Performed on Different Systems of $A_{2A}R$

System	N_{atoms}	N_{water}	N_{POPC}	Dimension($Å^3$)	Salt concentration	Simulation (ns)
NECA-bound cWT*-A_{2A}R-mini-$G\alpha_s\beta\gamma$	173,487	39,625	298	110.5*124.2*147.1	0.15M NaCl	2000*3
apo cR291A-mini-$G\alpha_s\beta\gamma$	173,420	39,622	298	110.5*124.2*147.1	0.15M NaCl	2000*3
apo cWT*-A_{2A}R-$G\alpha_s\beta\gamma$	191,445	43,079	339	115.1*115.1*155.8	0.15M NaCl	3000*3
apo cR291A-$G\alpha_s\beta\gamma$	191,349	43,053	339	115.1*115.1*155.8	0.15M NaCl	3000*3
