# Supplementary material for: Structure and function of a near fully-activated intermediate GPCR-Gαβγ complex
Source: Nat Commun. 2025 Jan 28;16:1100. doi: 10.1038/s41467-025-56434-4 (PMC11775185; doi:10.1038/s41467-025-56434-4)
Supplement: Supplementary file 2 — Description of Additional Supplementary Files [file 41467_2025_56434_MOESM2_ESM.pdf]

Description of Additional Supplementary Files

Supplementary Movie 1. 3D variability analysis (3DVA) of the entire particle set. The 3DVA movie highlights variability within the density, including motion along TM6, a slight lift of the H8 domain, and G protein movements. The models are referenced to the S4 conformation (Teal, PDB ID: 9EE8) and S4d1 conformation (Magenta, PDB ID: 9EE9).

Supplementary Movie 2. Dynamic motion in the S4 state. This movie depicts morphing from the $S4_{d1}$ conformation (Magenta, PDB ID: 9EE9) to the S4 conformation (Teal, PDB ID: 9EE8), highlighting noticeable swinging motions of the G protein while showing minimal changes in the receptor and its engagement with the G protein.

Supplementary Movie 3. Overview of the transition from the S4 state to the S5-mini-$G\alpha_s\beta\gamma$ complex. The movie illustrates the morphing transition from the S4 state (PDB ID: 9EE8) to the S5-mini- $G\alpha_s\beta\gamma$ state (PDB ID: 6GDG), showing receptor compaction and deeper insertion of the G protein into the receptor cavity.

Supplementary Movie 4. Interfacial transition involving the Ca5 helix of mini-Ga_s from the S4 to S5 state. This movie highlights interactions in the S4 state as an intermediate stage in the transition to the S5 state, focusing on the insertion of the Ca5 helix. It shows G protein compaction, insertion of the Ca5 helix of mini-Gas, and slight counterclockwise rotations of TM5, TM6, TM7, and H8.
