# Supplementary material for: Structure and function of a near fully-activated intermediate GPCR-Gαβγ complex
Source: Nat Commun. 2025 Jan 28;16:1100. doi: 10.1038/s41467-025-56434-4 (PMC11775185; doi:10.1038/s41467-025-56434-4)
Supplement: Supplementary file 8 — Transparent Peer Review file [file 41467_2025_56434_MOESM8_ESM.pdf]

nature portfolio

Peer Review File

Structure and function of a near fully-activated intermediate GPCR-Gαβγ complex

Corresponding Author: Dr Libin Ye

Parts of this Peer Review File have been redacted as indicated to remove third-party material.

This file contains all reviewer reports in order by version, followed by all author rebuttals in order by version.

Version 0:

Reviewer comments:

Reviewer #1

(Remarks to the Author)
The manuscript by Bi M. *et al*. characterizes the intermediate S4 state of adenosine A_{2A} receptor ($A_{2A}R$) using the conformation biased mutant R291A, which they have previously shown to trap the receptor in the S4 state. The authors found that the R291A mutant was able to bind Gαβγ with a decreased GTP hydrolysis and nucleotide exchange rate that is independent of ligand. Using both cryo-EM and Gaussian accelerated molecular dynamics simulation (GaMD), the authors determined the structure of the R291A $A_{2A}R$ in complex with mini-Gαβγ and proposed a mechanism by which the S4 state carry out the nucleotide exchange and transition to the fully activated state. This paper provides a great follow-up to the authors' previous paper on trapping the intermediate states of a GPCR using the mutants R291A and R293A. This work is interesting because the authors showed in atomic detail the orientation and the interactions of the relevant residues involved in the intermediate state. The results of this study increase our understanding on the features of the intermediate state and filled in the gap on the structural details of the GPCR activation landscape.

I have some comments that I think would help improve the paper if addressed:

Comments:

1. The authors found that the R291A-Gαβγ complex has a lower Michaelis-Menten constant Km for GTP hydrolysis than the fully activated state (WT*-Gαβγ-NECA). Can the authors elaborate on the implication of this?

2. On page 5, lines 141-142, the authors said that "BODIPY-FL-GTP showed more efficient binding to the S5-mediated G protein with a higher K_D value." What do the authors mean by this? In Figures 2d and 2e, based on the difference in K_D, it seems that R291A-Gαβγ has a stronger affinity toward GTP or GDP than the S5-mediated one.

3. I want to make sure that I understand it correctly. In Figure 2g, the intermediate state has a slightly faster rate of GDP exchange with GTP than the fully activated state, but since the fluorescence does not go to zero, it suggests that the BODIPY-FL-GDP is still bound, unlike the S5 state? Maybe the authors can include an additional statement explaining this observation on the text.

4. If I understand it correctly, the authors are trying to make a case that the 2nd association step observed in the BODIPY-FL-GDP binding assay with R291A is due to the limited access of the nucleotide to the second site by demonstrating that the association step is also observed with S5 state when BODIPY-FL-GTPγS is used since the bulky BODIPY group is on the γ-phosphate side. However, this wasn't conveyed very clearly in the main text. The authors may consider revising their statements in the manuscript to better communicate the argument.

5. When the nucleotide accessed the second site in the R291A-Gαβγ complex, the authors posit that the complex remains in the partially open S4 state. Did the authors try collecting the ^{19}F NMR spectrum of the R291A-Gαβγ complex with GDP or GTP to see if the receptor does not sample a different state?

6. Since the intermediate state of the A_{2A}R-Gαβγ complex can now be trapped and characterized, the application of this is that it is possible to design a therapeutic agent that can target this specific conformation of the receptor. Do the authors know of any disease or condition where this could be applied?

Minor Comments:
7. In the Methods section, GTPase hydrolysis assay, page 19, lines 436-442, change the description of the methods to passive voice.

8. Figure 2a, indicating which ligand is a full, partial, or inverse agonist on the figure would help the readers.

9. Figure 2c, the y-axis label is incorrect. I believe it should be velocity instead of GTP concentration.

10. Figure 2f, the legend label should be R291A+ Gαβγ and WT*+Gαβγ+NECA for consistency with the legends in the other panels.

11. The ordering of the panels in Figures 3 and 4 could be rearranged as they are referenced in the text. In the current version of the manuscript, after Fig. 3b, Fig. 3f is then referenced, followed by Fig. 3d, 3e, 3g, then Fig. 3c. Same with Figure 4, Fig. 4f is referenced, followed by Fig. 4e, 4d, 4g-i.

12. Extended Figure 2a, missing legend for red and teal data points.

13. Kindly provide clearer images for Extended Data Figures 3c and 3d.

14. Extended Figure 3b, the bands for A_{2A}R R291A and NB35 are not that visible on the SDS-PAGE gel for the most dominant peak (Fractions 18-20).

15. Extended Data Figure 4 caption: Schematic flow*chart*, instead of flow-*chat*.

16. The F376-M60 interaction was mentioned toward the end of the Results section, that it plays a significant role in the closed state of AHD, but this interaction was broken in both S4 and S5. Since this interaction is not a major key player in the transition between S4 and S5 states, Figures 4g, h, and I can probably be moved to the supplementary.

Reviewer #2

(Remarks to the Author)
Review of Bi et al.

The manuscript by Bi and coworkers reports the structure of mutant structure of A2A in complex with a mini-Gs. The aim of this work is to describe what the authors term an ‘intermediate’ transition state. This work was mostly guided by F19 NMR experiments where they observed an incomplete chemical shift to a fully active state structure. While the experiments are largely well conceived and performed, this reviewer has some fundamental issues with the interpretation of the data. I will say at the outset that I’m not a molecular dynamics expert and will not review that portion of the manuscript.

Firstly, I am absolutely not convinced that they have isolated a ligand free structure. In the maps the authors shared and one that I calculated (from their half maps), there is very clear density of something that hasn’t been modelled in the active site of their structure. It is clearly not a ligand free structure, in fact Adenosine perfectly fits in the unmodelled density.
It is not clear to me at all that the authors have isolated and solved a structure of an ‘intermediate’ S4 state. Their evidence that they have rests largely on their F19 NMR studies. While these NMR experiments are a first step in assessing the bulk chemical environments that the Fluorine samples in their different constructs it may not be the most robust way to determine that they have fully trapped an S4 state.

I’m also not sure the GTP exchange assays are fully formed. While there is clear phenotypic differences between their WT* and the R291A mutant, it is not clear to this reviewer that this is due to some intermediate state. As no G-protein association assays (BRET or FRET based) or G-protein turnover assays were performed this reviewer is concerned that the phenotypic differences could be more due to differential G-protein association rather than trapping an S4 state.

Furthermore, during the processing of the SPA data in their final 3D classification prior to consensus map refinement it appears that they may have ‘cherry-picked’ a particular dynamic state which may or may not reflect an S4 state. As all their 3D classes looked to be of high quality (2nd last row in Sup Figure 4), their final consensus reconstruction may not be reflective of the totality of states captured during the SPA experiment. Also, there was much discussion around relatively minor differences between their structure and another active state A2A structure, but the authors performed no 3D variability to assess the extent of dynamic states captured. In this reviewers opinion all the particles in the 3D classification step should be included in a 3DVA analysis (either cryoSPARC or cryoDRGN) to judge if their analysis of the PDB coordinates is justified or if they have simply captured one of many stable subsets of an active state structure. Even a comparison to their other 3D classes would be interesting to see if any of them look more like the active state structure that was compared against (6GBG).

To summarize, I'm not convinced that the authors have captured a ligand free structure and that they have captured an intermediate state structure. Much more analysis of their SPA data is warranted and perhaps some pharmacology type experiments to tease out what is going on with G-protein association to more robustly interpret their GTP experiments would be necessary to confirm the claims this manuscript makes.

Some minor issues:
1. Many side chains are unmodelled in the PDB, many of which have interpretable density (for example M177 and Y271 in receptor, to name just a few).
2. The Figure Legend in Figure 1 is mislabelled (d and f)
3. I don't know what the authors mean in Page 6. Line 183 "Closer inspection of the Tm6…. Using F19 … revealed a clockwise rotation…" This needs some explanation as I can't see what the authors mean here.
4. I would suggest to show the nucleotide exchange data for with the addition of agonists and inverse agonists for the mutant (maybe just in the sup material)
5. There are some more modelling errors. The unmodelled loop between 148 and 166 in the receptor will most likely have the N-terminus pointing in the wrong direction. As its currently modelled the only way for this loop to be completed is by forming a protein knot through ECL3.
6. This reviewer has major issues with the use of DeepEMhancer for modelling. The map that the authors originally provided is a prime example of AI hallucination. The AI model started to turn the detergent micelle into protein looking density and should NOT be trusted. The map I calculated from the half maps and the authors traditionally sharpened map is more than suitable for PDB modelling. EM enhanced maps also SHOULD NOT be the main map deposited in the PDB, as is taints the PDB and EMDB with data that is not suitable for interpretation.

Matthew Belousoff, Monash School of Pharmaceutical Sciences

Reviewer #3

(Remarks to the Author)
This manuscript reports the structure and function of a ligand-free GPCR-Gαsβγ intermediate complex. The authors determined the cryo-EM structure of an intermediate A2AR-mini-Gαsβγ complex. They presented experimental and computational evidences that the intermediate complex (S4-Gαsβγ) initiates a rate-limited nucleotide exchange without progressing to the fully activated complex (S5-Gαsβγ), in which the α-helical domain (AHD) of the Gαs is partially open engaged by a second nucleotide. Based on these, they proposed a mechanistic model for the rate-limited nucleotide exchange in the intermediate GPCR-Gαsβγ complex.
The experimental method presented in this paper is suitable and the MD simulations are well-designed. The approach can be applied to characterize transient intermediate states of other GPCRs. However, there are a few short comings in this study. I recommend that this work is publishable with revision as follows:
1. The authors determined the cryo-EM structure of an intermediate A2AR-mini-Gαsβγ complex. Has there any other intermediate state of other GPCRs reported in other papers? What's the similarity and difference between this intermediate structure and the others? It would be better to discuss this intermediate A2AR-mini-Gαsβγ structure in the context of the literature.
2. In the determined cryo-EM structure of the A2AR-mini-Gαsβγ complex, the AHD of Gαs was missing. The mini-Gαsβγ is different from the full-length Gs protein, will this influence the binding of Gs and A2AR?
3. It is seen from Fig.1a that the most obvious difference between the intermediate complex (S4-Gαsβγ) and other complex (S1-Gαsβγ, S2-Gαsβγ, S3-Gαsβγ and S5-Gαsβγ) is whether the AHD of Gs is partially open and whether there is GDP bound in Gs. It would be nice if the A2AR bound by the full-length Gs protein (with GDP bound in Gs) is characterized by cryo-EM.
4. The authors proposed two nucleotide-bound sites (Fig. 5, site 1 and site 2) in the transition process from the intermediate to the fully activated GPCR-Gαsβγ complex. They performed additional GaMD simulations to examine the GDP release process in the cS4-Gαsβγ system. GDP bound in site 2 of Gαsβγ in the starting structure of GaMD simulations of GDP release. In the simulation of the GDP release process, can the GDP be observed to make a short stay at site 1?
5. Free energy calculations indicated a "GDP Released" state in the S5-Gαsβγ system and only a "Partially Released" state in the S4-Gαsβγ system (Extended Data Fig. 8d and 8e). In this "Partially Released" state, is the GDP still binds in Gαsβγ? Is there a possibility that it binds in the proposed "site 1"?
6. Page 8 Lines 246: "Extended Data Figs. 8a-c". It should be Extended Data Figs. 8d-e.
Page 8 Lines 244-249: "Extended Data Figs. 8d-e". It should be Extended Data Figs. 8a-c.

Version 1:

Reviewer comments:

Reviewer #1

(Remarks to the Author)
The authors have addressed all my concerns and have updated the manuscript accordingly.

Some minor corrections:

1. Extended Data Figure 2, page 33: The figure caption seems to be mislabeled. I think the red data points represent the line width of S1 resonance and the teal data point represent the line width of the S4 resonance.

2. Page 5 lines 134-135: Omit the word *domain* after AHD

3. Page 5 line 134-135: formed by Ras-like domain and AHD resulting from a *partial opening* of AHD.

Reviewer #2

(Remarks to the Author)
Firstly, I wish to commend the authors on their significant efforts to reanalyse the cryoEM data. I am of the opinion that this has vastly improved the authors manuscript. I do not fundamentally disagree with the overall experimental design and overall analysis but I have a comment that the authors may wish to consider.

Our research group has performed 3DVA on over 300 GPCR ternary complexes and we observe incredibly similar dynamic sampling of both G-protein engagement angles and G-protein conformations. However, does this mean their interpretation is incorrect? I do not think so, it is more likely that they have found an appropriate experimental system to more fully explore and interpret the dynamics and 3DVA data that are commonly observed across all active state GPCR structures. Put another way, essentially all reported structures of active state GPCRs are an average of the S4-S5 state, but the authors in this manuscript have perhaps skewed the overall population towards the S4 state and provided a great hypothesis for interpretation of the observed dynamic landscape in active state GPCR structures.

I would recommend this paper for publication as it is my opinion that the authors have appropriately addressed the reviewers concerns.

Matthew Belousoff, Monash Institute of Pharmaceutical Sciences

Reviewer #3

(Remarks to the Author)
The authors have addressed all my previous concerns with detailed explanations and appropriate revisions. The revisions have improved the overall quality of the manuscript.I have no further issues with the current version of the manuscript, and I believe it can be published.

Open Access This Peer Review File is licensed under a Creative Commons Attribution 4.0 International License, which permits use, sharing, adaptation, distribution and reproduction in any medium or format, as long as you give appropriate credit to the original author(s) and the source, provide a link to the Creative Commons license, and indicate if changes were made.
In cases where reviewers are anonymous, credit should be given to 'Anonymous Referee' and the source.
The images or other third party material in this Peer Review File are included in the article’s Creative Commons license, unless indicated otherwise in a credit line to the material. If material is not included in the article’s Creative Commons license and your intended use is not permitted by statutory regulation or exceeds the permitted use, you will need to obtain

permission directly from the copyright holder.
To view a copy of this license, visit https://creativecommons.org/licenses/by/4.0/

Reviewer #1 (Remarks to the Author):

The manuscript by Bi M. *et al*. characterizes the intermediate S4 state of adenosine A_{2A} receptor ($A_{2A}R$) using the conformation biased mutant R291A, which they have previously shown to trap the receptor in the S4 state. The authors found that the R291A mutant was able to bind Gαβγ with a decreased GTP hydrolysis and nucleotide exchange rate that is independent of ligand. Using both cryo-EM and Gaussian accelerated molecular dynamics simulation (GaMD), the authors determined the structure of the R291A $A_{2A}R$ in complex with mini-Gαβγ and proposed a mechanism by which the S4 state carry out the nucleotide exchange and transition to the fully activated state. This paper provides a great follow-up to the authors' previous paper on trapping the intermediate states of a GPCR using the mutants R291A and R293A. This work is interesting because the authors showed in atomic detail the orientation and the interactions of the relevant residues involved in the intermediate state. The results of this study increase our understanding on the features of the intermediate state and filled in the gap on the structural details of the GPCR activation landscape.

Thanks for the appreciation on our new findings.

I have some comments that I think would help improve the paper if addressed:

Comments:

1. The authors found that the R291A-Gαβγ complex has a lower Michaelis-Menten constant Km for GTP hydrolysis than the fully activated state (WT*-Gαβγ-NECA). Can the authors elaborate on the implication of this?

The lower Km implies that the GTP-GDP conversion rate at the non-canonical nucleotide binding site 2 in the R291A-Gαβγ complex was much smaller than at the canonical site (site 1) in the WT*-$G\alpha_s\beta\gamma$-NECA, indicating a limited capacity of the intermediate complex in regulating GTP hydrolysis while the fully activated complex holds the full capacity. Of note, we redefine the canonical binding site as the site 1 while the noncanonical binding site as the site 2. This also implies that the GTP doesn't bind to the canonical nucleotide binding site in the R291A-Gαβγ complex.

2. On page 5, lines 141-142, the authors said that "BODIPY-FL-GTP showed more efficient binding to the S5-mediated G protein with a higher K_D value." What do the authors mean by this? In Figures 2d and 2e, based on the difference in K_D, it seems that R291A-Gαβγ has a stronger affinity toward GTP or GDP than the S5-mediated one.

We appreciate the reviewer's insight. Our interpretation is that the GTP binding site in the S5-mediated G protein is freely accessible because of the full open of AHD domain, resulting in a robust BODIPY-FL-GTP association-dissociation process. In contrast,

due to the space limitation caused by a partial open pose of AHD domain in the S4-mediated G protein, the binding of BODIPY-FL-GTP to the canonical nucleotide binding site is not accessible. The binding process is halted at the stage that Ras-like domain and AHD domain hold the nucleotide en route to the fully open state. We think this nucleotide binding site 2 is close to the canonical nucleotide binding site 1, which allows it to exhibit a limited GTP hydrolysis capacity, along with a slow dissociation rate as presented in the figures. This also results in the R291A-Gαβγ exhibiting a stronger affinity toward GTP or GDP than the S5-mediated one, as pointed out by the reviewer.

3. I want to make sure that I understand it correctly. In Figure 2g, the intermediate state has a slightly faster rate of GDP exchange with GTP than the fully activated state, but since the fluorescence does not go to zero, it suggests that the BODIPY-FL-GDP is still bound, unlike the S5 state? Maybe the authors can include an additional statement explaining this observation on the text.

Yes. The data in Fig. 2 indicated that the release number of BODIPY-FL-GDP from the S4 state was much less than the S5 state, but it reached the equilibrium much faster, resulting in the K_{off} actually 0.09/min vs 0.05/min (Page 5, lines 153-156). This was also consistent with our earlier findings in Figs. 2d and 2e showing that a substantial proportion of BODIPY-FL-GDP/GTP remained bound in the S4 state mediated G proteins.

4. If I understand it correctly, the authors are trying to make a case that the 2nd association step observed in the BODIPY-FL-GDP binding assay with R291A is due to the limited access of the nucleotide to the second site by demonstrating that the association step is also observed with S5 state when BODIPY-FL-GTPγS is used since the bulky BODIPY group is on the γ-phosphate side. However, this wasn't conveyed very clearly in the main text. The authors may consider revising their statements in the manuscript to better communicate the argument.

Yes, our conclusion is well articulated by the reviewer. We used the BODIPY-FL-GTP-γ-S as a case to confirm that the non-canonical binding site 2 is formed by the Ras-like domain and a partial open AHD domain that limits nucleotide to access canonical site 1. (Page 5, lines 148-152).

5. When the nucleotide accessed the second site in the R291A-Gαβγ complex, the authors posit that the complex remains in the partially open S4 state. Did the authors try collecting the ^{19}F NMR spectrum of the R291A-Gαβγ complex with GDP or GTP to see if the receptor does not sample a different state

Yes, the ^{19}F-NMR spectra were acquired with GDP included. We did see a small portion (~10%) of S3 state in the sample of R291A-Gαβγ while the receptor was unable to shift to the S5 state (Fig. 1b and Extended Data Fig.1).

6. Since the intermediate state of the A_{2A}R-Gαβγ complex can now be trapped and characterized, the application of this is that it is possible to design a therapeutic agent that can target this specific conformation of the receptor. Do the authors know of any disease or condition where this could be applied?

Thanks for the insights regarding the correlation between conformation and disease. So far, we didn't explore the details and the distinction of signaling pathways that S4 and S5 could respectively regulate, considering the complexity of signaling involved with various G proteins, GRKs, and β-arrestins. However, we acknowledge the potential for therapeutic applications and this type of work has been in our plan. In May 2024, a clinic variance R291C (NCBI, SNP: rs745714462). was reported, despite the functional data of this variance is still missing in the report. Considering the similarity of R291C and R291A from the molecular interaction level, we will continue and expand our study to use the knock-in mouse model to study the correlation between the conformation and disease in the future.

Minor Comments:
7. In the Methods section, GTPase hydrolysis assay, page 19, lines 436-442, change the description of the methods to passive voice.

We have revised the description in the GTPase hydrolysis assay section to passive voice to improve clarity and consistency. (Pages 21-22, lines 479-487)

8. Figure 2a, indicating which ligand is a full, partial, or inverse agonist on the figure would help the readers.

We have updated Fig. 2a to clearly indicate which ligands are full, partial, or inverse agonists, making it easier for readers to interpret the data. (Page 14, Fig. 2a)

9. Figure 2c, the y-axis label is incorrect. I believe it should be velocity instead of GTP concentration.

We have corrected the y-axis label in Fig. 2c to "velocity" for accuracy. (Page 14, Fig. 2c).

10. Figure 2f, the legend label should be R291A+ Gαβγ and WT*+Gαβγ+NECA for consistency with the legends in the other panels.

We have revised the legend in Fig. 2f for consistency with the other panels. (Page 14, Fig. 2f)

11. The ordering of the panels in Figures 3 and 4 could be rearranged as they are referenced in the text. In the current version of the manuscript, after Fig. 3b, Fig. 3f is

then referenced, followed by Fig. 3d, 3e, 3g, then Fig. 3c. Same with Figure 4, Fig. 4f is referenced, followed by Fig. 4e, 4d, 4g-i.

Thank you for pointing this out. A new figure was added before the previous version of Figure 3, resulting in a renumbering of figures. We have carefully reviewed the figure references and revised the order of the panels in Figs. 4 and 5 to match the sequence in which they are discussed in the text (Pages 8-10). This adjustment ensures better flow and alignment between the manuscript and the figures. We appreciate your feedback in helping to improve the clarity and organization of the manuscript.

12. Extended Figure 2a, missing legend for red and teal data points.

We appreciate you bringing this to our attention. The missing legend for the red and teal data points in Extended Data Fig. 2a has been added. (Page 33, Extended Data Fig. 2).

13. Kindly provide clearer images for Extended Data Figures 3c and 3d.

We have reprocessed and replaced Extended Data Figures 4c and 4d with higher-resolution images to improve clarity and ensure the details are more visible. (Page 35, Extended Data Fig.4).

14. Extended Figure 3b, the bands for $A_{2A}R$ R291A and NB35 are not that visible on the SDS-PAGE gel for the most dominant peak (Fractions 18-20).

The reduced visibility of the NB35 band was because we didn't heat the samples but merely incubated the sample with a loading buffer for better visibility of the receptor. This process could be the reason for the intensity decrease of soluble proteins like NB35. The same pattern was observed in others' reports as well, please refer to Response Fig. 1[1,2].

[*REDACTED*]

Response Figure 1. SDS-PAGE analysis of GPCR complex. a GPR52 receptor complex, including Gβγ, mini-Gα_s, and NB35 (Lin et al., 2020, Nature). **b** the purified components of the GLP-1R complex include Gα_s, Gβγ and NB35 (Cary et al., 2022, Nature Chemical Biology).

15. Extended Data Figure 4 caption: Schematic flow*chart*, instead of flow-*chat*.

Thank you for catching this typo. The caption for Extended Data Fig. 6 in the revised version has been corrected to "Schematic flowchart." (Page 37, Extended Data Fig. 6).

16. The F376-M60 interaction was mentioned toward the end of the Results section, that it plays a significant role in the closed state of AHD, but this interaction was broken in both S4 and S5. Since this interaction is not a major key player in the transition between S4 and S5 states, Figures 4g, h, and I can probably be moved to the supplementary.

Thank you for your suggestion. We have moved these panels to the supplementary and labeled them as Extended Data Figs. 12a, b, and c, as recommended.

Reviewer #2 (Remarks to the Author):

Review of Bi et al.

The manuscript by Bi and coworkers reports the structure of mutant structure of A2A in complex with a mini-Gs. The aim of this work is to describe what the authors term an 'intermediate' transition state. This work was mostly guided by F19 NMR experiments where they observed an incomplete chemical shift to a fully active state structure. While the experiments are largely well conceived and performed, this reviewer has some fundamental issues with the interpretation of the data. I will say at the outset that I'm not a molecular dynamics expert and will not review that portion of the manuscript.

1. Firstly, I am absolutely not convinced that they have isolated a ligand free structure. In the maps the authors shared and one that I calculated (from their half maps), there is very clear density of something that hasn't been modelled in the active site of their structure. It is clearly not a ligand free structure, in fact Adenosine perfectly fits in the unmodelled density.

We thank the reviewer for pointing this out. Our previous interpretation that the structure is ligand-free was based on experiments in which no exogenous ligands were added, and we did not consider the endogenous adenosine binding to the structure. We appreciate the reviewer's insight. Following his suggestion, we conducted additional analysis using a focused mask and confirmed that there is a clear extra density within the ligand-binding pocket, which can be modeled as adenosine. Subsequent mass spectrometry (Extended Data Fig.5 in the main text) confirmed the presence of adenosine in our sample. These findings suggest that the R291A mutant

stabilizes the receptor in a ligand-bound intermediate S4 state, distinct from the fully activated, ligand-bound S5 state. We have revised the text and figures accordingly and included the mass spectrometry result in the manuscript.

2. It is not clear to me at all that the authors have isolated and solved a structure of an 'intermediate' S4 state. Their evidence that they have rests largely on their F19 NMR studies. While these NMR experiments are a first step in assessing the bulk chemical environments that the Fluorine samples in their different constructs it may not be the most robust way to determine that they have fully trapped an S4 state.

With respect, we disagree with the reviewer's comment. ^{19}F NMR is a widely accepted and robust method for mapping conformational ensembles or landscapes. As demonstrated in our previous publication[3], the R291A mutation significantly enriched the population of the S4 state. While it is theoretically possible to capture intermediate states in SPA through classification alone, this specific mutation greatly enriches the S4 population, which in turn enables both detailed structural analysis and functional measurements of GTP hydrolysis at this intermediate state. In response to the reviewer's concern, we performed thorough classification, which revealed dynamic structural fluctuations centered around a predominant conformation, distinct from the well-defined S5 state. We interpret this predominant conformation as the S4 state, consistent with our ^{19}F NMR results.

As an orthogonal approach, we validated the existence of this intermediate S4 state through MD simulations, using the 6GDG structure with the R291A mutation and a full G-protein. These simulations, conducted independently of the cryo-EM data, confirmed a high structural similarity between the simulated S4 state and our cryo-EM structure. This provides further confidence that we have indeed captured the intermediate S4 state. We appreciate the reviewer's suggestion and acknowledge that the term "trapped" may have implied a more homogenous population than intended. We have revised the manuscript accordingly to clarify this.

3. I'm also not sure the GTP exchange assays are fully formed. While there is clear phenotypic differences between their WT* and the R291A mutant, it is not clear to this reviewer that this is due to some intermediate state. As no G-protein association assays (BRET or FRET based) or G-protein turnover assays were performed this reviewer is concerned that the phenotypic differences could be more due to differential G-protein association rather than trapping an S4 state.

We have conducted GTP turnover assays in our previous publication (Xudong Wang, et al. Nature Communications, 2023, Supplement Fig. 7a)[3]. We also attach the figure here (Response Fig. 2) as well as the current research (Figs. 2a-c) in addition to nucleotide exchange experiments (Figs. 2d-g). The experiments clearly showed that R291A mutant exhibited limited capacities of GTP turnover and nucleotide exchanges.

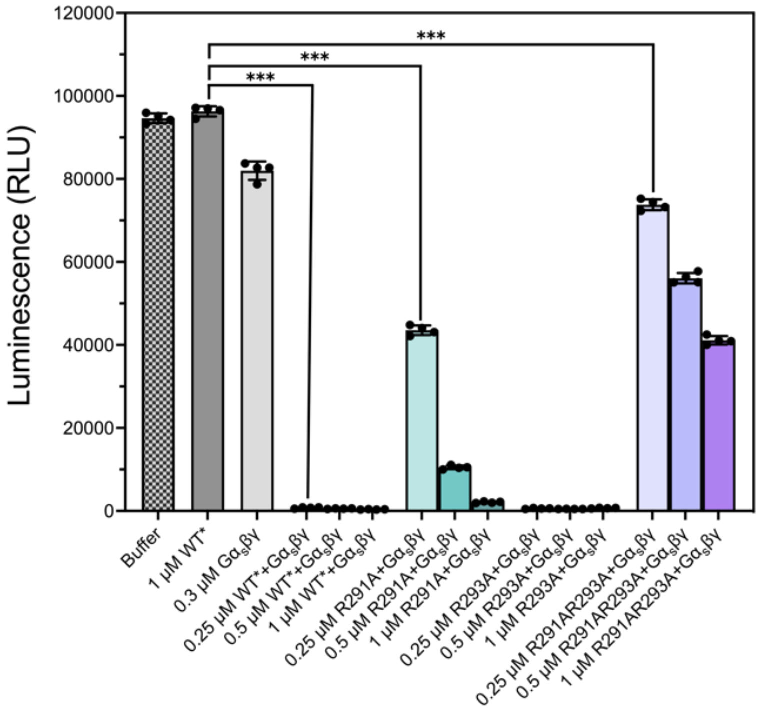

Response Figure 2. GTP hydrolysis assays for different mutants. GTP hydrolysis assays for WT* and mutants R291A (teal), R293A, and R291AR293A (violet) as a function of receptor concentrations from 0.25 μM to 1 μM. Of note, the references were normalized in two sets of measurements. Data with error bars are presented as mean±SEM of four independent experiments. Statistical analyses were performed using the ordinary oneway ANOVA followed the two-sides sidak's post-hoc test in PRISM 9.3.1, ***$p < 0.001$, in comparison to the WT*.

4. Furthermore, during the processing of the SPA data in their final 3D classification prior to consensus map refinement it appears that they may have 'cherry-picked' a particular dynamic state which may or may not reflect an S4 state. As all their 3D classes looked to be of high quality (2nd last row in Sup Figure 4), their final consensus reconstruction may not be reflective of the totality of states captured during the SPA experiment. Also, there was much discussion around relatively minor differences between their structure and another active state A2A structure, but the authors performed no 3D variability to assess the extent of dynamic states captured. In this reviewers opinion all the particles in the 3D classification step should be included in a 3DVA analysis (either cryoSPARC or cryoDRGN) to judge if their analysis of the PDB coordinates is justified or if they have simply captured one of many stable subsets of an active state structure. Even a comparison to their other 3D classes would be interesting to see if any of them look more like the active state structure that was compared against (6GBG). To summarize, I'm not convinced that the authors have captured a ligand free structure and that they have captured an intermediate state structure. Much more analysis of their SPA data is warranted and perhaps some

pharmacology type experiments to tease out what is going on with G-protein association to more robustly interpret their GTP experiments would be necessary to confirm the claims this manuscript makes.

We thank the reviewer for these comments, which led us to conduct a more detailed re-analysis of our data. In response, we revisited the entire particle set and all noted classes. Through extensive classification and 3D variability analysis (3DVA), we reconfirmed that the S4 conformation identified in our initial submission remains the predominant conformation. This conformation shows clear distinctions from the well-defined S5 state (6GDG). C-α displacement analysis across all classes indicated that the conformational snapshots cluster around this predominant S4 conformation, with most fluctuations occurring in the Gγ subunit. This finding is consistent with our ^{19}F NMR data, which revealed an enrichment centered around a defined peak, further supporting the identification of S4 as a distinct intermediate state on the trajectory to full activation. Additional receptor pocket calculations are provided in Response Fig.3.

We focused on two snapshots that show the largest deviations from the predominant S4 conformation, which we refer to as $S4_{d1}$ and $S4_{d2}$. The remaining snapshots exhibit only minor variations in the G protein, while in $S4_{d1}$, the outermost region of the G protein swings by up to 7.5 Å compared to the S4 state, likely due to intermediate G-protein engagement. However, the receptor itself displays only subtle variations within the overall S4 conformation. We have updated the text and figures to include these findings and have illustrated the conformational similarities among the classes using per-residue C-α displacement calculations, as shown in Extended Data Fig. 9 (Page 40).

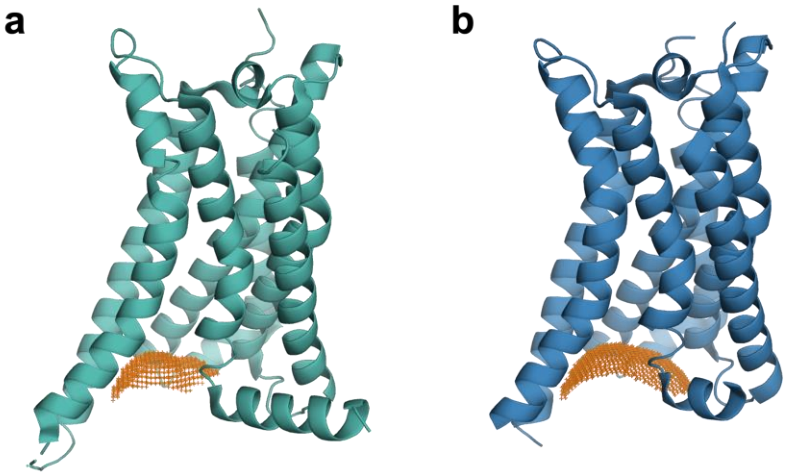

Response Figure 3. Cavity volume comparison between S4 and S5 states using KVFinder. a the cavity in structure A (cyan) representing the S4 state has a volume of 293.33 Å³. **b** the cavity in structure B (blue) representing the S5 state shows a larger volume of 534.38 Å³. Calculations were performed using probes with inner and outer radii of 5 Å and 10 Å, respectively.

Some minor issues:
1. Many side chains are unmodelled in the PDB, many of which have interpretable density (for example M177 and Y271 in receptor, to name just a few).
Thank you for pointing this out. We have now modeled the side chains for M177 and Y271, and we have manually inspected all relevant residues to ensure the best fit. This includes adding residues such as V164, A165, among others.

2. The Figure Legend in Figure 1 is mislabelled (d and f)
Thank you for your careful inspection. We have corrected the figure legend for Figure 1.

3. I don't know what the authors mean in Page 6. Line 183 "Closer inspection of the Tm6…. Using F19 … revealed a clockwise rotation…" This needs some explanation as I can't see what the authors mean here.
We have revised the wording on page 8, lines 222-226 for clarity. We used the ^{19}F-tag labeling site as a reference to track conformational changes and to illustrate our ^{19}F-qNMR data. The ^{19}F probe reveals a clockwise rotation as the receptor transitions towards activation, with the NMR signal for the S4 state appearing at a lower field compared to the S5 state, as shown in Fig. 4c.

4. I would suggest to show the nucleotide exchange data for with the addition of agonists and inverse agonists for the mutant (maybe just in the sup material)

Thank you for the suggestion. We have indeed performed nucleotide exchange experiments with the addition of both agonists and inverse agonists for the mutant. The data has been included in the revised manuscript and can be found in the supplementary material (Extended Data Fig. 3).

5. There are some more modelling errors. The unmodelled loop between 148 and 166 in the receptor will most likely have the N-terminus pointing in the wrong direction. As its currently modelled the only way for this loop to be completed is by forming a protein knot through ECL3.

Thank you for your detailed inspection. We were able to model V164, A165 into the density map, which provided a clear direction for the loop between residues 147-164. Additionally, I corrected the fitting errors for F70 and C71. The updated model now clearly shows that the loop is not forming a knot through ECL3.

6. This reviewer has major issues with the use of DeepEMhancer for modelling. The map that the authors originally provided is a prime example of AI hallucination. The AI model started to turn the detergent micelle into protein looking density and should NOT be trusted. The map I calculated from the half maps and the authors traditionally sharpened map is more than suitable for PDB modelling. EM enhanced maps also

SHOULD NOT be the main map deposited in the PDB, as is taints the PDB and EMDB with data that is not suitable for interpretation.

We used DeepEMhancer map solely for illustration and initial modeling purposes. However, we have confirmed that all model refinements in Phenix were conducted using the raw map without the use of DeepEMhancer. For each EMDB and PDB entry, we have deposit both the sharpened map (sharpened by -10Å in cisTEM) and the raw unsharpened map. In the revised manuscript, we have removed all references to the DeepEMhancer map.

Matthew Belousoff, Monash School of Pharmaceutical Sciences

Reviewer #3 (Remarks to the Author):

This manuscript reports the structure and function of a ligand-free GPCR-Gαsβγ intermediate complex. The authors determined the cryo-EM structure of an intermediate A2AR-mini-Gαsβγ complex. They presented experimental and computational evidences that the intermediate complex (S4-Gαsβγ) initiates a rate-limited nucleotide exchange without progressing to the fully activated complex (S5-Gαsβγ), in which the α-helical domain (AHD) of the Gαs is partially open engaged by a second nucleotide. Based on these, they proposed a mechanistic model for the rate-limited nucleotide exchange in the intermediate GPCR-Gαsβγ complex.

Thanks for the commendation on our manuscript.

The experimental method presented in this paper is suitable and the MD simulations are well-designed. The approach can be applied to characterize transient intermediate states of other GPCRs. However, there are a few short comings in this study. I recommend that this work is publishable with revision as follows:
1. The authors determined the cryo-EM structure of an intermediate A2AR-mini-Gαsβγ complex. Has there any other intermediate state of other GPCRs reported in other papers? What's the similarity and difference between this intermediate structure and the others? It would be better to discuss this intermediate A2AR-mini-Gαsβγ structure in the context of the literature.

Thank you for the thoughtful suggestion. While no intermediate complex of $A_{2A}R$ has been reported so far, a partially active structure has been reported with the binding of LUF5833 and LUF5834[4,5]. In response to the reviewer's suggestion, we have added a discussion of other GPCR intermediate states in the literature to contextualize our findings, please refer to page 11, lines 318-328.

Additionally, we compare our structure determined in this manuscript with all resolved

receptor-Gs complex for family A GPCR with the assistance of MD simulation (Fig.4c and 4d). Our results clearly indicate the uniqueness of our structure representing an intermediate position from the G protein perspective on both Ras-like domain (resolved structure) and AHD domain (simulated structure).

2. In the determined cryo-EM structure of the A2AR-mini-Gαsβγ complex, the AHD of Gαs was missing. The mini-Gαsβγ is different from the full-length Gs protein, will this influence the binding of Gs and A2AR?

We agree that using the mini-Gα_sβγ instead of the full-length Gs protein may influence binding. However, obtaining an intermediate GPCR-G protein complex with the full-length Gs protein remains challenging. We believe that protein-engineering of the G protein, similar to what we did for the receptor, would help stabilize an intermediate G protein conformation, and this is part of our future plans.

3. It is seen from Fig.1a that the most obvious difference between the intermediate complex (S4-Gαsβγ) and other complex (S1-Gαsβγ, S2-Gαsβγ, S3-Gαsβγ and S5-Gαsβγ) is whether the AHD of Gs is partially open and whether there is GDP bound in Gs. It would be nice if the A2AR bound by the full-length Gs protein (with GDP bound in Gs) is characterized by cryo-EM.

Thank you for the suggestion. As mentioned above, we agree that characterizing the full-length Gs protein in complex with $A_{2A}R$ would provide more insight. This is part of our ongoing research, and we plan to explore this by engineering a stable full-length G protein in future studies.

4. The authors proposed two nucleotide-bound sites (Fig. 5, site 1 and site 2) in the transition process from the intermediate to the fully activated GPCR-Gαsβγ complex. They performed additional GaMD simulations to examine the GDP release process in the cS4-Gαsβγ system. GDP bound in site 2 of Gαsβγ in the starting structure of GaMD simulations of GDP release. In the simulation of the GDP release process, can the GDP be observed to make a short stay at site 1?

Thank you for your detailed analysis. As shown in Fig. 5c, GDP rapidly transitions from the bound to the unbound state, with no intermediate state observed during this process. Similarly, as indicated in Extended Data Fig. 11e, no other low-energy states were detected. Additionally, the initial state of the G protein starts in a fully open conformation, and during GDP release, it remains largely open rather than adopting a partially open state. This could be the caveat of the MD here to capture the nucleotide binding to the site 2 (we redefine the original site 2 as the site 1 and the original site1 as site 2) that was observed in our bench experiments. We will modify our model in the future or waiting for the structure of an intermediate $A_{2A}R$-full length G protein resolved to revisit this phenomenon.

5. Free energy calculations indicated a “GDP Released” state in the S5-Gαsβγ system and only a “Partially Released” state in the S4-Gαsβγ system (Extended Data Fig. 8d and 8e). In this “Partially Released” state, is the GDP still binds in Gαsβγ? Is there a possibility that it binds in the proposed “site 1”?

The representative structure of the 'Partially Released' state from the current research is shown below (Response Fig. 4). In this state, the phosphate group remains bound within the binding site, interacting with residues G52, S54, T55, and K293. Currently, we lack the data to confirm whether this corresponds to the proposed 'site 2.' Further studies will be conducted to investigate this hypothesis as mentioned above till we have the intermediate A_{2A}R-full length G protein complex and using it as a starting model for simulation.

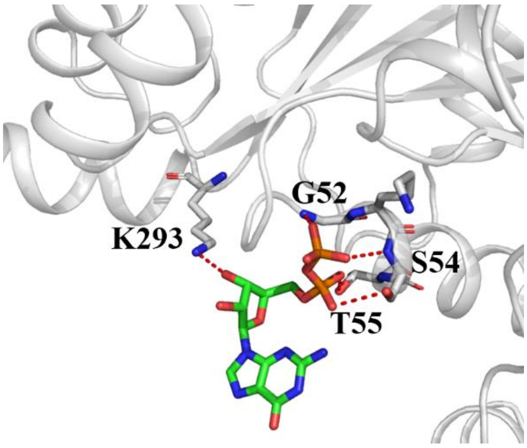

Response Figure 4. Representative conformation of the 'Partially Released' state is depicted. Key interacting residues are shown in stick representation, with red dotted lines indicating their interactions.

6. Page 8 Lines 246: “Extended Data Figs. 8a-c”. It should be Extended Data Figs. 8d-e.Page 8 Lines 244-249: “Extended Data Figs. 8d-e”. It should be Extended Data Figs. 8a-c.
Thank you for pointing out these discrepancies. We have corrected the references to the figures (Page 10, line 286 and line 289).

References:

1 Cary, B. P. *et al.* Structural and functional diversity among agonist-bound states of the GLP-1

receptor. *Nat. Chem. Biol* **18**, 256-263 (2022). https://doi.org/10.1038/s41589-021-00945-w

2 Lin, X. *et al.* Structural basis of ligand recognition and self-activation of orphan GPR52. *Nature* **579**, 152-157 (2020). https://doi.org/10.1038/s41586-020-2019-0

3 Wang, X., Neale, C., Kim, S.-K., Goddard, W. A. & Ye, L. Intermediate-state-trapped mutants pinpoint G protein-coupled receptor conformational allostery. *Nat. Commun.* **14**, 1325 (2023). https://doi.org/10.1038/s41467-023-36971-6

4 Claff, T. *et al.* Structural Insights into Partial Activation of the Prototypic G Protein-Coupled Adenosine A2A Receptor. *ACS Pharmacol. Transl. Sci.*. **7**, 1415-1425 (2024). https://doi.org/10.1021/acsptsci.4c00051

5 Amelia, T. *et al.* Crystal Structure and Subsequent Ligand Design of a Nonriboside Partial Agonist Bound to the Adenosine A2A Receptor. *J. Med. Chem.* **64**, 3827-3842 (2021). https://doi.org/10.1021/acs.jmedchem.0c01856

We would like to thank all reviewers for their positive responses to our previous revision. In this final revision, we address all the remaining comments.

Reviewer #1 (Remarks to the Author):

The authors have addressed all my concerns and have updated the manuscript accordingly.

Some minor corrections:

1. Extended Data Figure 2, page 33: The figure caption seems to be mislabeled. I think the red data points represent the line width of S1 resonance and the teal data point represent the line width of the S4 resonance.

Thanks for pointing out this and we have corrected all these accordingly.

2. Page 5 lines 134-135: Omit the word *domain* after AHD

We have omitted the word “domain” after AHD and corrected all these throughout the manuscript.

3. Page 5 line 134-135: formed by Ras-like domain and AHD resulting from a *partial opening* of AHD.
We have changed the sentence as the reviewer suggested.

Reviewer #2 (Remarks to the Author):

Firstly, I wish to commend the authors on their significant efforts to reanalyse the cryoEM data. I am of the opinion that this has vastly improved the authors manuscript. I do not fundamentally disagree with the overall experimental design and overall analysis but I have a comment that the authors may wish to consider.

Our research group has performed 3DVA on over 300 GPCR ternary complexes and we observe incredibly similar dynamic sampling of both G-protein engagement angles and G-protein conformations. However, does this mean their interpretation is incorrect? I do not think so, it is more likely that they have found an appropriate experimental system to more fully explore and interpret the dynamics and 3DVA data that are commonly observed across all active state GPCR structures. Put another way, essentially all reported structures of active state GPCRs are an average of the S4-S5 state, but the

authors in this manuscript have perhaps skewed the overall population towards the S4 state and provided a great hypothesis for interpretation of the observed dynamic landscape in active state GPCR structures.

We appreciate this comment. Indeed, it is likely that our approach of combining 19F NMR and point mutation to bias the conformation towards S4 enabled us to characterize this intermediate state more thoroughly. We revised the discussion to reflect this point.

I would recommend this paper for publication as it is my opinion that the authors have appropriately addressed the reviewers concerns.

We thank Dr. Belousoff for his support in publishing our manuscript and appreciate his comments, which helped us improve it.

Matthew Belousoff, Monash Institute of Pharmaceutical Sciences

Reviewer #3 (Remarks to the Author):

The authors have addressed all my previous concerns with detailed explanations and appropriate revisions. The revisions have improved the overall quality of the manuscript. I have no further issues with the current version of the manuscript, and I believe it can be published.

We thank the Reviewer for his/her support.
